# Diurnal Changes in Transcript and Metabolite Levels during the Iron Deficiency Response of Rice

**DOI:** 10.1186/s12284-017-0152-7

**Published:** 2017-04-20

**Authors:** Jamie Selby-Pham, Adrian Lutz, Laura T. Moreno-Moyano, Berin A. Boughton, Ute Roessner, Alexander A. T. Johnson

**Affiliations:** 1 School of BioSciences, The University of Melbourne, Parkville, Victoria Australia; 2 Metabolomics Australia, The University of Melbourne, Parkville, Victoria Australia

**Keywords:** LC-MS, DMA, Strategy II, Plant stress response, Metal stress

## Abstract

**Background:**

Rice (*Oryza sativa* L.) is highly susceptible to iron (Fe) deficiency due to low secretion levels of the mugineic acid (MA) family phytosiderophore (PS) 2′-deoxymugineic acid (DMA) into the rhizosphere. The low levels of DMA secreted by rice have proved challenging to measure and, therefore, the pattern of DMA secretion under Fe deficiency has been less extensively studied relative to other graminaceous monocot species that secrete high levels of PS, such as barley (*Hordeum vulgare* L.).

**Results:**

Gene expression and metabolite analyses were used to characterise diurnal changes occurring during the Fe deficiency response of rice. Iron deficiency inducible genes involved in root DMA biosynthesis and secretion followed a diurnal pattern with peak induction occurring 3–5 h after the onset of light; a result consistent with that of other Strategy II plant species such as barley and wheat. Furthermore, triple quadrupole mass spectrometry identified 3–5 h after the onset of light as peak time of DMA secretion from Fe-deficient rice roots. Metabolite profiling identified accumulation of amines associated with metal chelation, metal translocation and plant oxidative stress responses occurring with peak induction 10–12 h after the onset of light.

**Conclusion:**

The results of this study confirmed that rice shares a similar peak time of Fe deficiency associated induction of DMA secretion compared to other Strategy II plant species but has less prominent daily fluctuations of DMA secretion. It also revealed metabolic changes associated with the remediation of Fe deficiency and mitigation of damage from resulting stress in rice roots. This study complements previous studies on the genetic changes in response to Fe deficiency in rice and constitutes an important advance towards our understanding of the molecular mechanisms underlying the rice Fe deficiency response.

**Electronic supplementary material:**

The online version of this article (doi:10.1186/s12284-017-0152-7) contains supplementary material, which is available to authorized users.

## Background

Iron (Fe) is utilized in a variety of essential biological processes such as the respiratory (Park et al. 1996) and photosynthetic (Hung et al. 2010) electron-transport chain reactions (Connolly and Guerinot 2002; Reddy 2006), due to its stable redox properties, specifically an ability to donate and accept electrons between both Fe^+2^ (ferrous) and Fe^+3^ (ferric) redox states. Although Fe is the fourth most abundant element in the earth’s crust, it is not readily available to soil growing plants under aerobic conditions due to its tendency to form insoluble Fe^3+^ precipitates (Kim and Guerinot 2007). Insufficient Fe supply to plants leads to Fe depletion of both the mitochondria (Mori et al. 1991) and chloroplast (Stocking 1975; Terry and Low 1982), resulting in impaired efficiency of both cellular respiration and photosynthesis. Additionally, as Fe is required for the formation of the chlorophyll precursor δ-aminolevulinic acid (Marsh et al. 1963; Miller et al. 1984), Fe deficiency results in the characteristic yellowing or chlorosis of the youngest leaves (Terry and Low 1982). Furthermore, Fe deficiency is costly from an agronomic perspective (Hell and Stephan 2003) accounting for more than 25% of the crop yield losses of cereals, legumes and vegetables (Briat et al. 2015).

Iron is also the functional cofactor for the heme containing hemoprotein superfamily of enzymes involved in photosynthesis, respiration, DNA synthesis (Michel and Pistorius 2004) and antioxidants including peroxidases (Zaharieva and Abadía 2003). In cells, antioxidants quench reactive oxygen species (ROS) which are produced as an inevitable consequence of photosynthesis (Parida and Das 2005) and to a lesser extent due to respiration (Gill and Tuteja 2010). Cellular stress induced by Fe deficiency results in impaired antioxidant activity due to absence of the required catalytic Fe, leading to an increased abundance of cellular ROS (Becana et al. 1998; Iturbe-Ormaetxe et al. 1995). Accordingly Fe homeostasis must be tightly regulated in plants to prevent Fe-deficiency-induced oxidative damage to proteins and lipids (Sun et al. 2007).

Plants have evolved mechanisms to increase Fe uptake in response to conditions of limited Fe bioavailability. Dicots and non-graminaceous monocots employ Strategy I uptake, involving proton (H^+^) secretion to the rhizosphere and reduction of soil Fe^3+^ to Fe^2+^. Secretion of H^+^ reduces soil pH and promotes the solubility of Fe ions and reduction of Fe^3+^ to the more bioavailable Fe^2+^ promotes increased uptake of Fe^2+^ through iron-regulated transporters (IRT) in the plasma membrane of plant root cells (Lee & An 2009; Scholz et al. 1992). Graminaceous plant species (grass family Gramineae) utilize Strategy II uptake involving secretion of mugineic acid (MA) family phytosiderophores (PS) into the rhizosphere to chelate and solubilise the soil Fe^3+^, allowing for subsequent uptake of Fe^3+^-PS complexes through yellow stripe 1-like (YSL) transporters in the plasma membrane of plant root cells (Inoue et al. 2009; Nagasaka et al. 2009).

Rice (*Oryza sativa* L.) is not only the model monocot species but also a staple food that contributes 35–59% of the daily caloric intake for approximately three billion people (Bhullar and Gruissem 2013). Rice is often grown in flooded paddies where microorganisms rapidly deplete dissolved oxygen levels, resulting in anoxic water where Fe has the tendency to be reduced to the more bioavailable Fe^2+^ form (Walker and Connolly 2008). Rice has therefore evolved the unusual ability amongst other Strategy II plant species to absorb Fe^2+^ via OsIRT transporters in a manner similar to Strategy I plant species (Bughio et al. 2002; Ishimaru et al. 2006). However, whilst genome sequencing demonstrates that the rice genome contains ferric reductase genes (Vasconcelos et al. 2004), rice does not show ferric reductase activity in roots nor the characteristic swelling of roots in response to Fe deficiency as seen in Strategy I plant species (Rong-li et al. 2012). The utilization of aspects from both Strategy I and Strategy II Fe uptake within the rice Fe deficiency response is commonly referred to as the ‘Combined Strategy’ (Ishimaru et al. 2006; Sperotto et al. 2012). The *OsIRT* homologous genes *HvIRT1* in barley and *ZmIRT1* in maize have been reported to be upregulated in response to Fe deficiency. Further, expression of these genes in Fe uptake-defective yeast mutants has been demonstrated to reverse growth defect associated with Fe uptake inability (Li et al. 2013; Pedas et al. 2008). However, *in planta* uptake of Fe^2+^ by Strategy II plants has so far only been confirmed in rice using tracer experiments (Ishimaru et al. 2006; Ricachenevsky and Sperotto 2014).

The amount of PS secreted by Strategy II plants corresponds with plant tolerance to growth on alkaline soils (soils with low Fe bioavailability) as identified by resistance to the onset of Fe-deficiency-associated chlorosis (Marschner et al. 1986). In response to Fe limiting conditions, barley (*Hordeum vulgare* L.), a plant species able to grow vigorously on high pH soils, secretes large amounts of PS including 2′-deoxymugineic acid (DMA) and the derivatives MA and 3-epihydroxymugineic acid (epiHMA). By contrast, rice, which is highly susceptible to Fe deficiency chlorosis on high pH soils, secretes only DMA in response to Fe deficiency and at much lower levels and shorter durations than barley (Higuchi et al. 1996; Mori et al. 1991). During Fe deficiency, barley (Marschner et al. 1986; Mori et al. 1987; Takagi et al. 1984; Walter et al. 1995), wheat (Oburger et al. 2014; Zhang et al. 1991), maize (Ueno et al. 2009), and red fescue (Ma et al. 2003) secrete PS following a diurnal pattern, with higher levels of PS secreted during day (light) periods and lower levels of PS secreted during night (dark) periods.

As Fe is a cofactor in enzymes involved in photosynthesis, this secretion pattern may enable plant Fe uptake to coincide with increased photosynthetic rates during light periods (Takagi et al. 1984). Additionally, as PS are readily degraded by soil microbes as a carbon and/or nitrogen source, the diurnal pattern of PS release may help reduce the degree of PS loss by microbial populations in the rhizosphere (Römheld and Marschner 1990). During Fe deficiency, rice induces expression of DMA biosynthetic genes following a diurnal pattern (Inoue et al. 2009; Nozoye et al. 2004, 2011). Additionally, rice secretes higher levels of DMA in the morning than afternoon or night periods (Nozoye et al. 2014). However, the diurnal pattern of transcript and metabolite levels within the rice Fe deficiency response has not been as extensively investigated as the Fe deficiency response of barley.

We studied the diurnal changes of DMA, NA, transcripts and metabolite levels within the rice Fe deficiency response. Rice roots exposed to Fe deficiency were used to investigate diurnal changes in expression levels of genes involved in Strategy II Fe uptake and translocation. A highly sensitive detection method was utilized to quantify *in planta* NA and DMA levels as well as DMA secretion levels by Fe-deficient rice roots. Additionally, metabolite profiling of Fe-deficient root tissues was performed to understand the molecular mechanisms utilized within the rice Fe deficiency response.

## Results

### Induction of Fe Deficiency Symptoms

**Fig. 1:**
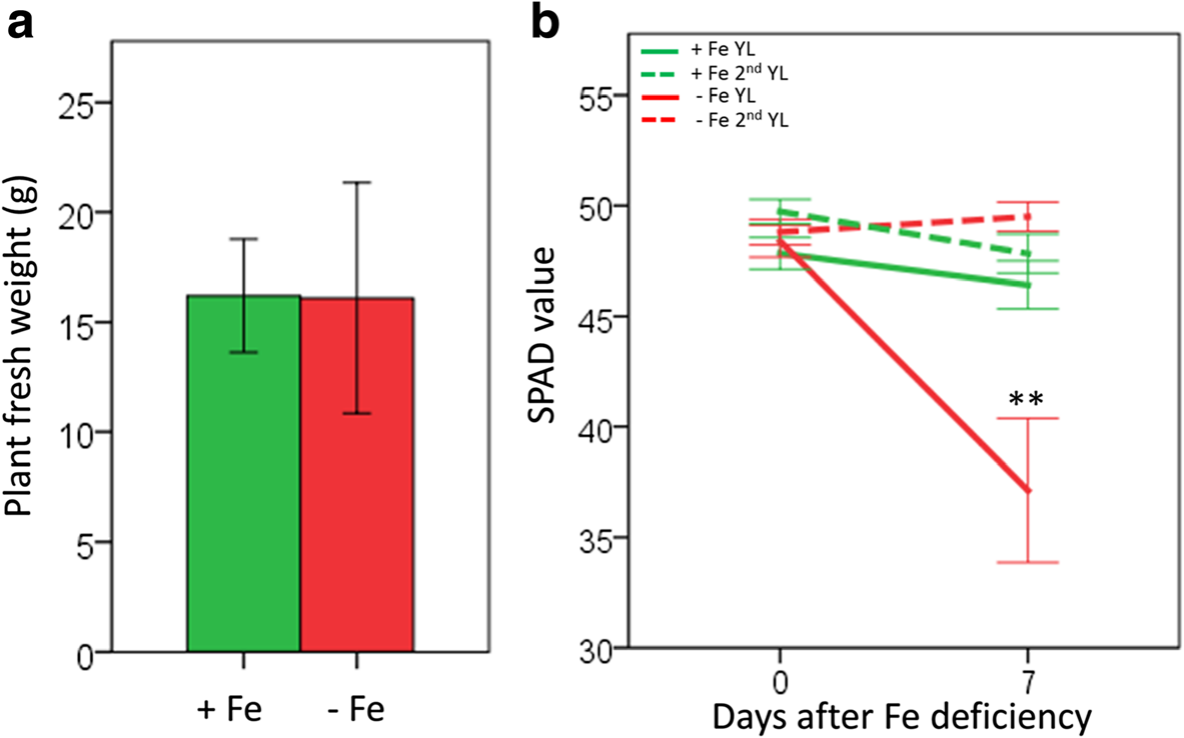
Induction of Fe deficiency symptoms in rice plants. **a** Plant fresh weight (*n* = 9). **b** SPAD values of the youngest leaf (YL) and 2^nd^ youngest leaf (2^nd^ YL) (*n* = 30). Plant fresh weights and leaf SPAD values of Fe-sufficient (*green*) and Fe-deficient (*red*) plants were measured at 0 and 7 days after the Fe deficiency treatment. Values are presented as means ± SE of *n* biological replicates. Significant differences are indicated by ** for *p* < 0.01 by Student’s t-tests performed between treatments within leaf types and days after Fe deficiency

Soil Plant Analysis Development (SPAD) measurements of leaf tissues identified that exposure of 5 week old hydroponically grown rice plants to one week of Fe deficiency induced a significant reduction in the youngest leaf ‘greenness’ (*p* ≤ 0.001); however, no significant variation in plant fresh weight was detected (*p* = 0.972). Accordingly, this treatment was selected as a means of inducing Fe deficiency symptoms in rice without affecting biomass (Fig. 1).

### Expression of Genes Involved in the Strategy II Response in Fe-deficient Rice Roots

**Fig. 2:**
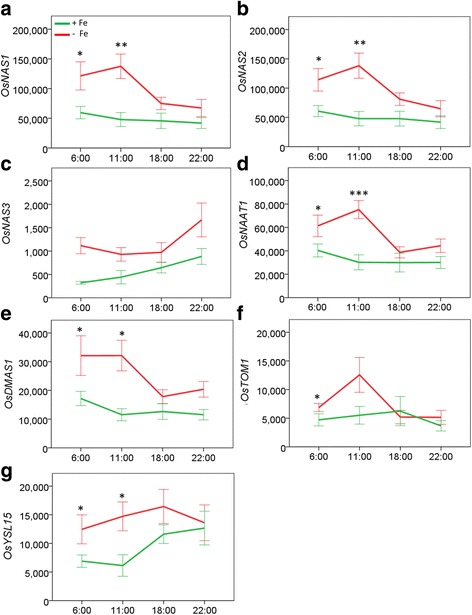
Expression of genes associated with DMA biosynthesis in Fe-sufficient and Fe-deficient rice roots. Expression of **a** *OsNAS1*, **b** *OsNAS2*, **c** *OsNAS3*, **d** *OsNAAT1*, **e** *OsDMAS1*, **f** *OsTOM1* and **g** *OsYSL15* genes in the roots of Fe-sufficient (*green*) and Fe-deficient (*red*) rice plants. Data is presented as means of genes of interest (GOI) expression relative to the three gene normalization factor (3GNF) ± SE of 9 biological replicates. Significant differences are indicated by * for *p* < 0.05, ** for *p* < 0.01, *** for *p* ≤ 0.001 by Student’s t-tests performed between treatments within collection times

Analysis of root transcripts (Additional file 1: Table S1A) by quantitative real time PCR (qRT-PCR) identified that Fe deficiency resulted in a significant increase in the expression of *OsNAS1* (*p* ≤ 0.001), *OsNAS2* (*p* ≤ 0.001), *OsNAS3* (*p* ≤ 0.001), *OsNAAT1* (*p* ≤ 0.001), *OsDMAS1* (*p* ≤ 0.001), and *OsYSL15* (*p* = 0.006), but no significant change in the expression of *OsTOM1* (*p* = 0.054). Additionally, a statistically significant diurnal pattern of expression during Fe deficiency was detected for *OsNAS1* (*p* = 0.016), *OsNAS2* (*p* = 0.017), *OsNAAT1* (*p* ≤ 0.001), *OsDMAS1* (*p* = 0.049) and *OsTOM1* (*p* = 0.012). Expression of *OsNAS1*, *OsNAS2*, *OsNAAT1*, *OsDMAS1* and *OsTOM1* were increased by Fe deficiency 1.5–2.0 fold during 6:00–8:00 and 2.3–2.9 fold during 11:00–13:00 (Additional file 1: Table S1B). In general, expression of these genes followed a diurnal pattern during Fe deficiency but not during Fe sufficiency (Fig. 2), wherein the peak of Fe deficiency induced expression occurred during 11:00–13:00. Accordingly, Fe deficiency promoted increased expression of genes involved in nicotianamine (NA) and DMA biosynthesis, DMA secretion, and uptake of Fe^3+^-DMA complexes following a diurnal pattern with a peak time of induction 3–5 h after the onset of light.

### Biosynthesis and Secretion of DMA in Fe-deficient Rice Roots

**Fig. 3:**
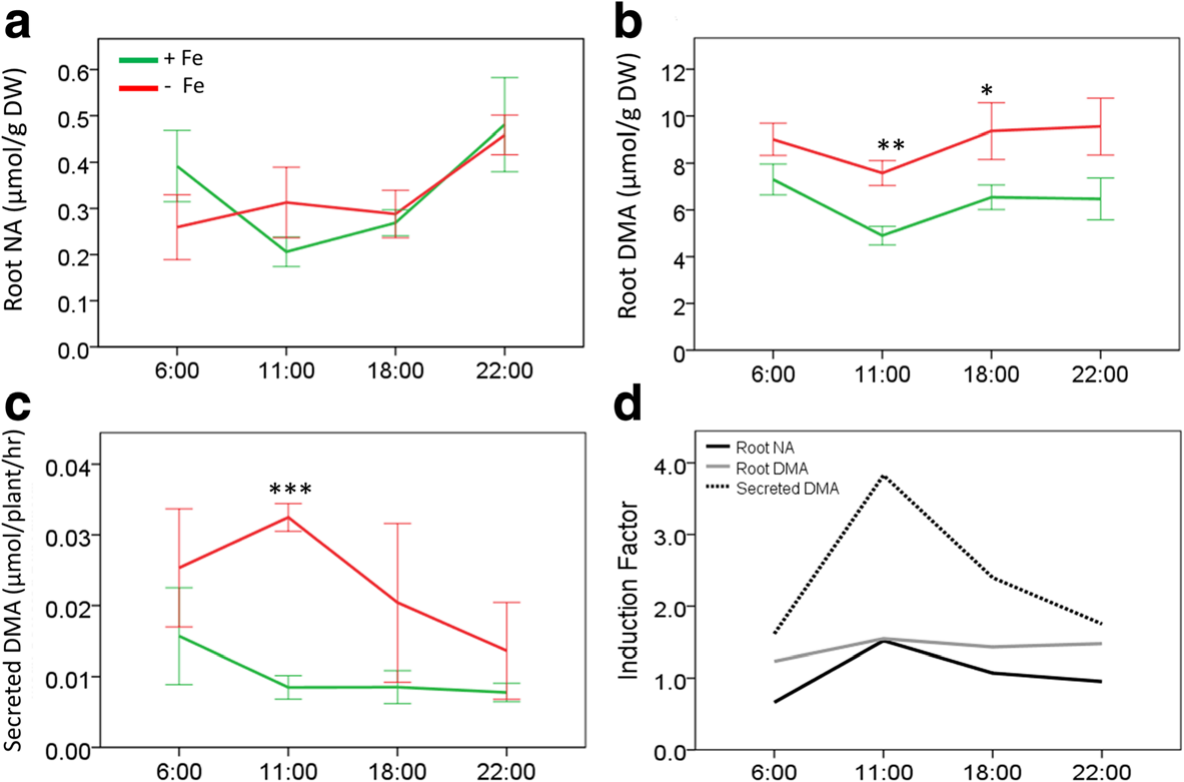
Concentration of NA, DMA and secreted DMA of Fe-sufficient and Fe-deficient rice roots. Root concentrations of **a** NA (*n* = 5), **b** DMA (*n* = 5) and **c** DMA secretion (*n* = 3) from Fe-sufficient (*green*) and Fe-deficient (*red*) rice roots were quantified by LC-MS. **d** Induction factors of root NA, DMA and secreted DMA concentrations. Values are presented as means ± SE of *n* biological replicates. Significant differences are indicated by * for *p* < 0.05, ** for *p* < 0.01, *** for *p* ≤ 0.001 as determined by Student’s t-tests performed between treatments within collection times

Targeted quantification of NA and DMA by liquid chromatography mass spectrometry (LC-MS) from root metabolite extracts collected after one week of Fe deficiency identified that concentrations of DMA (average 1.42-fold higher, *p* ≤ 0.001), but not NA (average 1.05-fold higher, *p* = 0.988), were significantly higher in the Fe-deficient roots (Fig. 3a and b, Additional file 2: Table S2A). Root concentrations of NA and DMA in Fe-sufficient plants showed a similar diurnal pattern, with root NA (*p* = 0.016) and DMA (*p* = 0.033) concentrations significantly higher during dark periods than light periods. While NA and DMA concentrations in Fe-deficient roots did not follow a significant diurnal pattern, Fe deficiency induction of root NA and DMA concentrations were both highest during the 11:00–13:00 period (Fig. 3d).

Quantification of DMA by LC-MS from root secretions collected after one week of Fe deficiency, identified that Fe-deficient roots secreted on average 2.4-fold more DMA (*p* = 0.009) than Fe-sufficient roots across the day (Fig. 3c). Whilst no significant diurnal variation was observed in DMA secretion by Fe-sufficient roots, a diurnal pattern with a peak during 11:00–13:00 (*p* = 0.001) was observed for DMA secretion by Fe-deficient roots. Accordingly, Fe deficiency promoted increased secretion of DMA following a diurnal pattern with a peak occurring 3–5 h after the onset of light.

### Metabolite Profiles of Fe-deficient Rice Roots

**Fig. 4:**
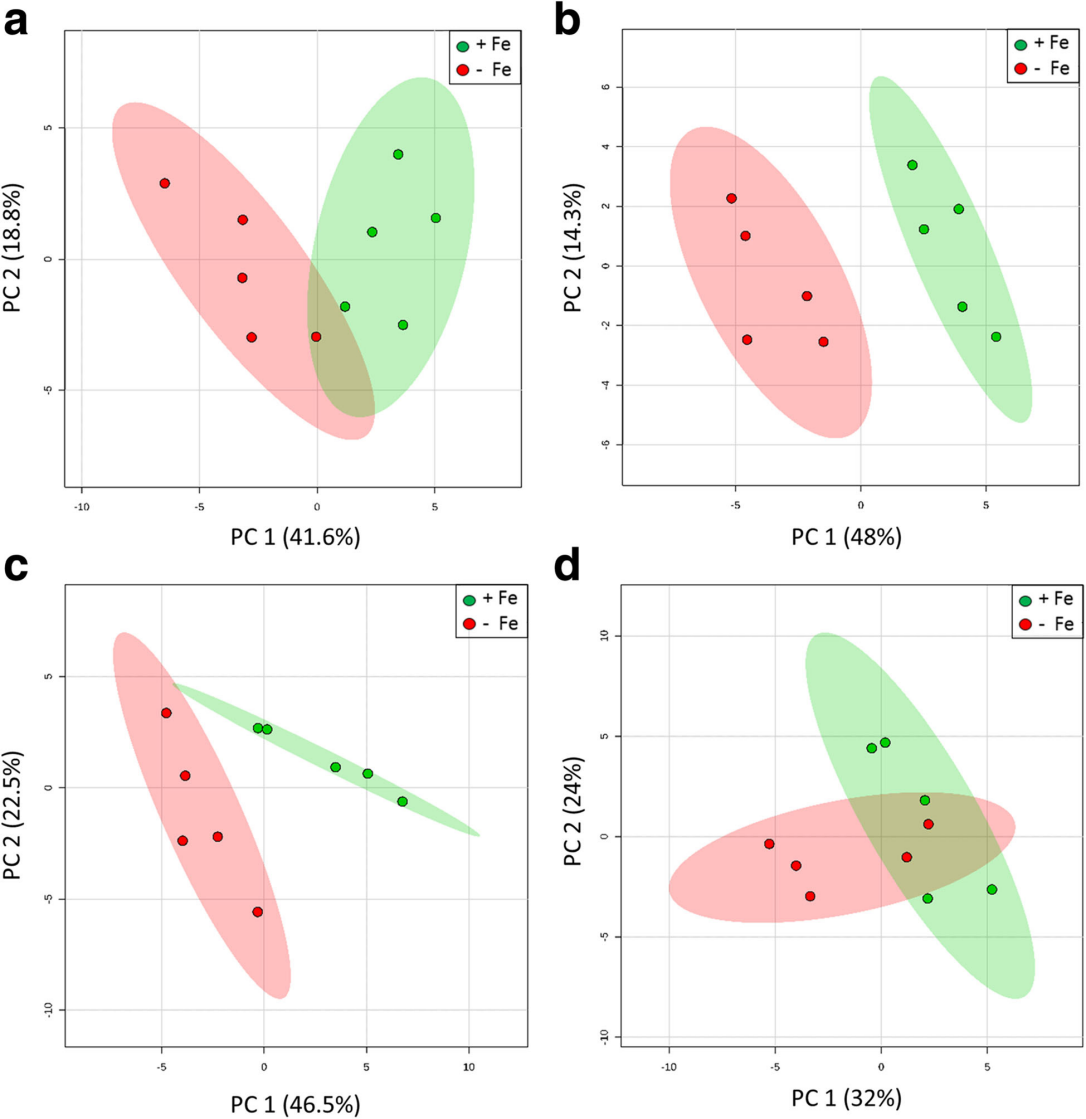
Principal component analyses of root metabolite profiles from Fe-sufficient and Fe-deficient rice roots. Metabolite profiles from Fe-sufficient *(+Fe: green*) and Fe-deficient (*−Fe: red*) rice roots were compared using 95% confidence regions within the collection times **a** 6:00–8:00, **b** 11:00–13:00, **c** 18:00–20:00 and **d** 22:00–24:00

Root tissue metabolite extracts were analysed by LC-MS to develop amines profiles (Additional file 2: Table S2A). PERMANOVA analyses showed highly significant differences in metabolite profiles between Fe treatments (Pseudo-*F* = 14.197, *p* = 0.001) and collection times (Pseudo-*F* = 2.494, *p* = 0.003). Principal component analysis (PCA) of root amines profiles (Fig. 4) identified clearly distinct 95% confidence regions between Fe-sufficient and Fe-deficient profiles for samples collected during the 11:00–13:00 time period only (Fig. 4b). However, significant differences in metabolite profiles between Fe treatments were observed for the collection times 6:00–8:00 (*t* = 1.983, *p* = 0.011), 11:00–13:00 (*t* = 2.610, *p* = 0.002), and 18:00–20:00 (*t* = 2.521, *p* = 0.008). No significant differences between metabolite profiles of different Fe treatments were observed during the 22:00–24:00 collection time (*t* = 1.562, *p* = 0.059). These findings indicate that the rice Fe deficiency response promoted changes to root metabolite profiles following a diurnal pattern with significant changes during 6:00–20:00 and a peak of Fe deficiency associated changes occurring during 11:00–13:00.

### Biochemical Pathway Mapping of the Rice Fe Deficiency Response

Concentrations of metabolites in the following pathways were significantly increased in the roots of Fe-deficient rice: α–ketoglutarate family amino acids, 3-phosphoglycerates (3-P-glycerates), pyruvate derivatives, aromatic amino acids, citrulline–nitric oxide (Cit-NO) cycle, oxaloacetate/aspartate family amino acids and the DMA biosynthetic pathway (Additional file 2: Table S2B). No metabolites were detected at significantly lower concentrations in the roots of the Fe-deficient rice.

**Fig. 5:**
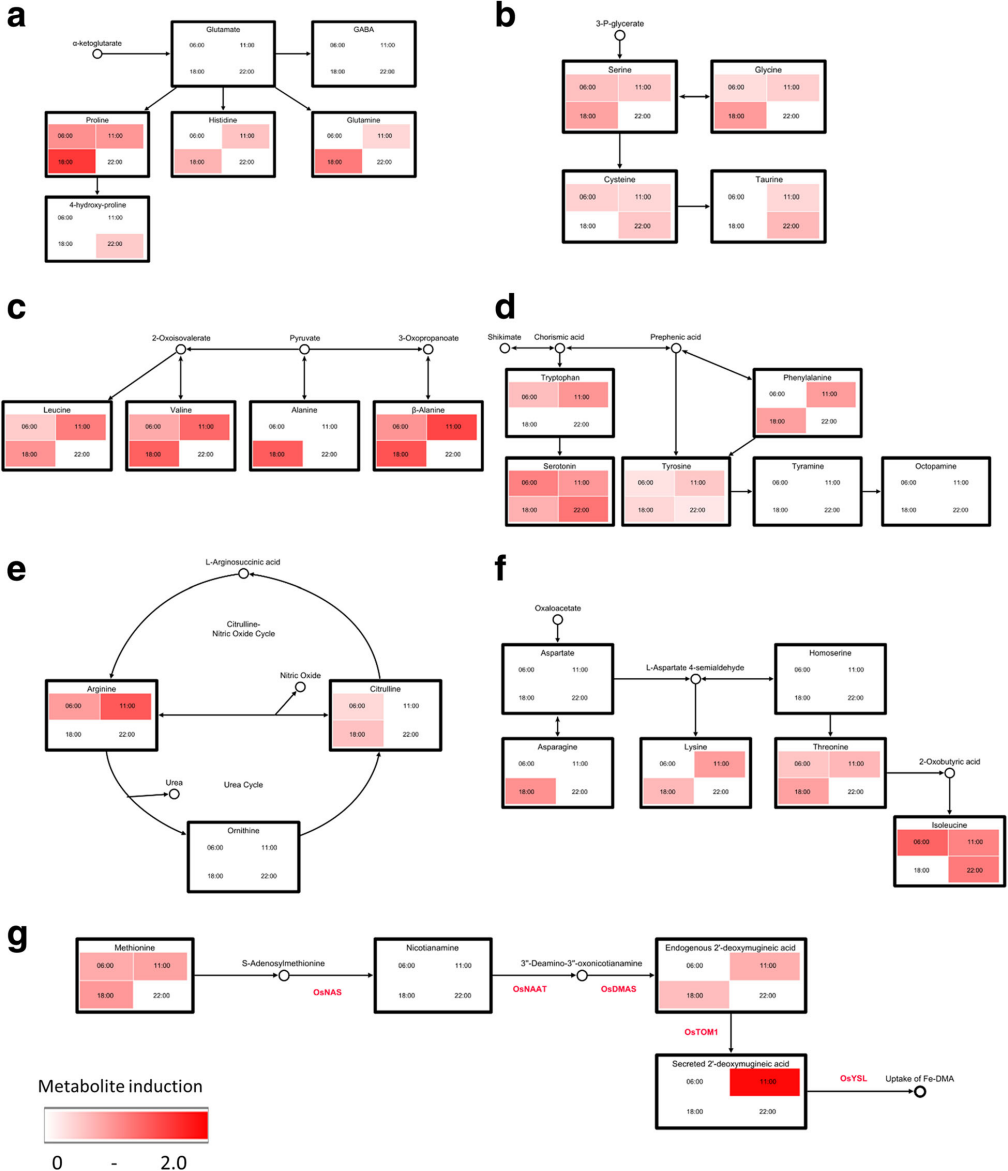
Changes in metabolic pathways induced by Fe deficiency in rice roots. Heatmap (ranging from 0–2.0 as *white*–*red*) of log_2_ transformed Fe deficiency associated induction of metabolite concentrations in biochemical pathways of **a** α-ketoglutarates, **b** 3-P-glycerates, **c** pyruvates, **d** aromatic amines, **e** Cit-NO/urea cycle, **f** aspartate family amino acids and **g** DMA biosynthesis and secretion

Induction factors associated with Fe deficiency were mapped onto custom biochemical pathways (Fig. 5) using the Visualization and Analysis of NeTworks with related Experimental Data program (VANTED) v2.1.0 (Plant Bioinformatics Group, The Leibniz-Institute of Plant Genetics and Crop Plant Research, available at https://immersive-analytics.infotech.monash.edu/vanted/). Induced levels of the α–ketoglutarate family amino acids (proline, histidine and glutamine) were observed in Fe-deficient roots following a diurnal pattern with a peak of induction occurring during 18:00–20:00 (Fig. 5a). Induced levels of the 3-P-glycerate derivatives serine and glycine (Fig. 5b) and pyruvate derivatives: valine, leucine, alanine and β-alanine (Fig. 5c) were also observed in Fe-deficient roots, suggesting an increase in glycolysis during light periods (11:00–13:00 and 18:00–20:00). Increased activity of glycolysis due to Fe deficiency was also supported by a significant increase in concentration of the glucose-to-pyruvate intermediate 3-P-glycerate in Fe-deficient roots (Additional file 3: Figure S1). Induced levels of the aromatic amino acids: phenylalanine, serotonin, tyrosine, and tryptophan in Fe-deficient roots (Fig. 5d), suggest an increased activity of the shikimate pathway. Induced levels of arginine and citrulline but not ornithine in Fe-deficient roots (Fig. 5e), suggest an increased activity of the citrulline–nitric oxide (Cit-NO) cycle. Additionally, induced levels of the oxaloacetate/aspartate family amino acids: isoleucine, lysine, threonine (Fig. 5f) and methionine (Fig. 5g) were detected during 6:00–8:00, 11:00–13:00 and 18:00–20:00, but not 22:00–24:00. Activity of the DMA biosynthetic pathway was also increased in Fe-deficient roots based on induced levels of methionine (the dominant precursor of DMA biosynthesis), root DMA and secreted DMA (Fig. 5g). Additionally, the potential methionine precursors serine and cysteine (but not aspartate or homoserine) accumulated in response to Fe deficiency (Additional file 4: Figure S3).

## Discussion

### Iron Deficiency Induces Expression of Genes Involved in Strategy II Fe Uptake Following a Diurnal Pattern

Detailed analysis of rice gene expression related to NA and DMA biosynthesis, as well as DMA secretion under Fe deficiency, has been described in several previous investigations (Inoue et al. 2003; Nozoye et al. 2011; Wang et al. 2013). Here we have identified diurnal patterns of expression for the *OsNAS1*, *OsNAS2*, *OsNAAT1*, *OsDMAS1*, and *OsTOM1* genes in Fe-deficient rice roots with significant induction at the 6:00 and 11:00 sampling times (Fig. 2, Additional file 1: Table S1). These diurnal patterns are consistent with previous investigations (Nozoye et al. 2004, 2011), and may be summarised as a slight induction of expression prior to the onset of light, a peak of induction 3–5 h after the onset of light, and a return to expression levels similar to that of Fe-sufficient roots prior to dark. While the diurnal patterns reported in this study have been previously identified, other studies have reported much higher inductions ratios for Fe-deficiency-inducible genes including *OsNAS1* and *OsNAS2* (Inoue et al. 2003; Kobayashi et al. 2009). This may have been due to differences in growing conditions, age of plants and length of exposure to the Fe deficiency treatment. In this study we cultivated rice plants in Fe-sufficient hydroponic solution for 5 weeks before transferring to Fe-deficient solution for a period of 1 week prior to sampling. Inoue et al. (2003) exposed very young seedlings to Fe-sufficient nutrient solution for 2 weeks, whereas Kobayashi et al. (2009) cultivated seedlings in Fe-sufficient nutrient solution for 3 weeks, before transferring plants to Fe-deficient solution for 1 week. Considering that our plants were more established than these studies prior to Fe deficiency, it is possible that our six week old plants were not fully Fe limited at the time of sampling and this may have reduced the induction ratios that we observed. We also found that expression of *OsYSL15* was significantly induced in response to Fe deficiency prior to the onset of light and 3–5 h after the onset of light (Fig. 2g, Additional file 1: Table S1A). However, the diurnal pattern of *OsYSL15* in Fe-deficient roots differed from the diurnal pattern of *OsNAS1, OsNAS2, OsNAAT1, OsDMAS1* and *OsTOM1* by having a peak time of expression at 18:00–20:00 instead of 11:00–13:00 (Fig. 2). OsYSL15 is an Fe-regulated Fe^3+^-DMA transporter and is thought to be responsible for active uptake of Fe^3+^-DMA complexes during daylight hours (Inoue et al. 2009). Thus, its expression may be induced by the preferential biosynthesis of root DMA at night time (Fig. 3b) and coordinated by diurnal secretion of DMA (Fig. 3c). Whilst increased expression of *OsYSL15* after the onset of light has been reported previously (Inoue et al. 2009), the diurnal pattern of *OsYSL15* that we observed is not consistent with the daily fluctuations of *OsYSL15* described by Inoue et al. (2009). This inconsistency could be explained by differences in rice genotype as well as experimental design. In this study we used rice cultivar Nipponbare whereas Inoue et al. (2009) utilized cultivar Tsukinohikari. These genotypic differences may influence *OsYSL15* expression under Fe deficiency. Inoue et al. (2009) also characterised the induction of expression of *OsYSL15* under Fe deficiency over a 27-h time period with tissue sampling intervals of 3 h, compared to our analysis over a 24-h time period with tissue sampling intervals of 4–7 h.

### Iron Deficiency Promotes DMA Biosynthesis and Accumulation

In this study we observed significant upregulation of *OsNAS1*, *OsNAS2*, *OsNAAT1* and *OsDMAS1* under Fe-deficient conditions (Fig. 2), however, only DMA (not NA) concentrations were significantly increased in the Fe-deficient rice roots (Fig. 3a and b); a result that is consistent with previous studies in barley and rice (Higuchi et al. 2001). These results imply that *OsNAS* upregulation within the Strategy II Fe deficiency response is associated with increased NA production for DMA biosynthesis, rather than NA accumulation. These results also suggest that increased DMA, rather than NA, is utilized for the altered chelation of Fe in the roots of Fe-deficient rice. This is supported by predictive models indicating that at the rice root apoplastic pH of 4.9 (Nishiyama et al. 2008), DMA would outcompete NA as the predominant Fe complex (Hider et al. 2004). Recent speciation studies of metal ligands within chelation complexes in the xylem and phloem sap of rice have identified that DMA is a major chelator of Fe in the phloem sap (Nishiyama et al. 2012; Yoneyama et al. 2015), but neither DMA nor NA are major chelators of Fe in the xylem sap (Ariga et al. 2014). Accordingly DMA-mediated translocation of Fe within the rice Fe deficiency response may occur predominantly via the phloem.

Whilst NA and DMA concentrations in Fe-sufficient roots showed a distinct diurnal pattern with higher concentrations in dark periods than light periods, no significant diurnal pattern of NA or DMA concentrations were observed in Fe-deficient roots. In barley, a diurnal pattern is observed wherein root PS (DMA, MA and epiHMA) concentrations are reduced during periods of heightened PS secretions (Kawai et al. 1993; Nagasaka et al. 2009; Walter et al. 1995). In the current study, Fe deficiency induced a 1.5-fold increase in root DMA concentrations and a 2.4-fold increase in DMA secretions in rice plants (Fig. 3), accounting for root DMA concentrations changing minimally during periods of increased DMA secretion. By contrast in barley, the depletion of root PS concentrations may be explained by Fe deficiency inducing an 8.7-fold increase in root PS concentrations but up to a 250-fold increase in PS secretions (Walter et al. 1995). This difference may relate to the Combined Strategy utilized by rice grown in flooded conditions involving uptake of unbound Fe^2+^ via IRT transporters. As this mechanism of Fe uptake is not reliant on DMA secretion, rice may have evolved to retain a larger proportion of the increased DMA produced during Fe deficiency.

### Iron Deficiency Promotes Increased Secretion of DMA Following a Diurnal Pattern

Successful modification of the DMA quantification method described in Kakei et al. (2009) from quadrupole time-of-flight mass spectrometry to the more sensitive triple quadrupole mass spectrometry provided a 3-fold improvement in sensitivity. Accordingly the developed DMA quantification method was more sensitive than DMA quantification methods described in previous publications including: Fe solubilisation assay (Rong-li et al. 2012), high performance liquid chromatography (Nozoye et al. 2014), LC-MS (Oburger et al. 2014), capillary electrophoresis mass spectrometry (Dell’mour et al. 2010) and nuclear magnetic resonance spectroscopy (Ma et al. 1995). This improved sensitivity allowed avoidance of the false-negative result wherein DMA within rice root secretions was deemed ‘not detected’ as the concentration was below the method’s limit of detection as in previous publications utilizing nuclear magnetic resonance spectroscopy for analysis (Fan et al. 2001).

Previous studies have identified that rice secretes more DMA during the 6:00–13:00 ‘morning’ period than the 13:00–20:00 ‘afternoon’ or 20:00–6:00 ‘night’ periods (Nozoye et al. 2014). Consistent with this result, our study identified that the peak time of DMA secretions by Fe-deficient rice occurred during the 6:00–13:00 ‘morning’ period (Fig. 3). Additionally, the improved sensitivity of the developed method allowed for the analysis of root secretions collected during the ‘morning’ over two narrower time periods (6:00–8:00 and 11:00–13:00). The ability to analyse root secretion DMA concentrations collected over narrower time periods allowed for further characterisation of rice DMA secretions during this ‘morning’ period leading to the identification of 11:00–13:00 (3–5 h after the onset of light) as the peak time of DMA secretion from Fe-deficient rice roots (Fig. 3).

Whilst this diurnal pattern of the Fe deficiency response in rice is less distinct than that of other graminaceous plants, the peak time of DMA secretions by rice is consistent with the peak time of PS secretions observed under Fe deficiency in barley (Marschner et al. 1986; Mori et al. 1987; Takagi et al. 1984; Walter et al. 1995), wheat (Oburger et al. 2014; Zhang et al. 1991), maize (Ueno et al. 2009), and red fescue (Ma et al. 2003). Consistent with other graminaceous plant species, a major aspect of the rice Fe deficiency response appears to involve re-establishing Fe homeostasis via increased PS mediated Fe uptake and internal translocation with the time of peak secretions occurring 3–5 h after the onset of light.

### Iron Deficiency in Rice Induces Changes to Carbon and Energy Metabolism

Previous proteome analyses have identified that the molecular mechanisms induced within plant Fe deficiency responses include changes to plant metabolism associated with: carbon and energy metabolism, S-adenosyl-methionine (SAM) metabolism, ion transport, ROS scavenging and cell signalling (Kosová et al. 2011). Proteome analyses of the rice Fe deficiency response specifically identified increased levels of the glycolytic enzyme glyceraldehyde-3-phosphate dehydrogenase in the roots of Fe-deficient rice (Chen et al. 2015). Results of the current study indicated increased glycolysis and respiration within the Fe deficiency response during light periods (11:00–20:00) based on the induced levels of 3-P-glycreate derivatives, branched chain amino acids, pyruvate derivatives (Fig. 5), and 3-P-glycerate (Additional file 3: Figure S1). This is consistent with previous studies on plant stress responses (Di Martino et al. 2003; Wingler et al. 2000; Zoghlami et al. 2011). The rice Fe deficiency response therefore involves changes to carbon and energy metabolism potentially to accommodate the increased energy demand required to drive mechanisms induced within the Fe deficiency response.

Methionine is the dominant precursor of DMA biosynthesis (Mori and Nishizawa 1987) via the intermediate SAM, which is derived from methionine by SAM synthetase within the methionine cycle (Suzuki et al. 2006). Transcript analysis of barley suggested that methionine and SAM biosynthesis were induced in response to Fe deficiency (Nagasaka et al. 2009; Negishi et al. 2002). Similarly, transcript analyses of rice suggested that activity of the methionine cycle and DMA biosynthesis are induced 24 h after the onset of Fe deficiency (Itai et al. 2013). In the current study, Fe-deficient rice roots showed induced levels of methionine during 6:00–20:00 (Fig. 5g). However, unlike the diurnal patterns of gene expression detected (Fig. 2), a clear peak of methionine concentrations was not observed during 11:00–13:00. Serine (via cysteine) and/or aspartate (via homoserine) are used by plants to produce homocysteine, the immediate precursor to methionine (Droux et al. 1995). Serine and cysteine levels were induced in Fe-deficient rice roots whilst levels of aspartate and homoserine were unchanged (Fig. 5b and f; Additional file 4: Figure S4). The co-accumulation of serine, cysteine and methionine suggested that serine may be the dominant precursor to the increased methionine biosynthesis within the rice Fe deficiency response. To confirm this hypothesis, further investigations will be required such as radioisotope tracer experiments previously used to confirm that methionine is the dominant precursor of PS biosynthesis within the Fe deficiency responses of graminaceous plants (Mori and Nishizawa 1987; Nakanishi et al. 1999).

### Iron Deficiency in Rice Induces Molecular Mechanisms Associated with Plant Metal-stress Responses

Ion transport, chelation and sequestration are key mechanisms involved in plant metal tolerance and reestablishment of metal homeostasis (Clemens 2001). In response to Fe deficiency, induced levels of histidine, proline and asparagine were detected during 18:00–20:00 (Fig. 5a and f). These amines have been identified as chelators of copper, nickel and zinc and facilitate tolerance to these metal stresses via complexation and xylem-mediated translocation (Rai 2002; Sharma and Dietz 2006). Considering that DMA is able to bind a range of metals in the rhizosphere (Scholz et al. 1992), and Fe-deficient rice has previously been shown to uptake increased concentrations of calcium, cadmium and zinc (da Silveira et al. 2007; Zhang et al. 1998), the increased DMA secretions during 11:00–13:00 within the rice Fe deficiency response may promote increased uptake of micronutrients other than Fe. The induced levels of chelators (histidine, proline, asparagine and DMA) during 18:00–20:00 within the rice Fe deficiency response may be associated with increased chelation and translocation of off-target metals (other than Fe) during the 11:00–13:00 period due to increased DMA secretions.

### Iron Deficiency in Rice Induces Molecular Mechanisms Involved in Mediation of Oxidative Stress

The increased concentration of ROS from abiotic stresses, including Fe deficiency, results in an “oxidative burst” which in-turn causes damage to cell proteins, lipids, carbohydrates and DNA (Gill and Tuteja 2010; Spinelli et al. 2011). Accordingly, the plant Fe deficiency response includes the induction of molecular mechanisms associated with mediation of the damage due to oxidative stress. Accumulation of amino acids within abiotic stress responses play a protective role as antioxidants which detoxify ROS and prevent damage to the plant cell (Joshi et al. 2010). The antioxidant proline was induced in roots of our Fe-deficient rice plants with a peak of induction occurring during 18:00–20:00 (Fig. 5a). This peak time may be associated with off-target metal uptake as the localized increase to metal concentrations may cause oxidative stress via formation of ROS (Ercal et al. 2001). Iron-deficient rice roots also showed induced levels of phenylalanine, tyrosine, serotonin and tryptophan (Fig. 5d) which are antioxidant aromatic amines (Nimalaratne et al. 2011; Ramakrishna and Ravishankar 2011; Stadtman and Levine 2003). Particularly, phenylalanine is the main precursor to a suite of phenolic compounds which have very high antioxidant activity (Ghasemzadeh and Ghasemzadeh 2011; Michalak 2006).

Iron deficiency was associated with a significant decrease in root accumulation of ascorbic acid (Additional file 3: Figure S2), which is typically the primary polar antioxidant in plants (Weber et al. 2008). Previous proteomic studies have identified decreased levels of ascorbate peroxidase in the roots of Fe-deficient rice (Chen et al. 2015). As ascorbate peroxidase is a hemoprotein, Fe deficiency results in a decrease in ascorbate peroxidase activity (Becana et al. 1998; Iturbe-Ormaetxe et al. 1995; Sun et al. 2007). Additionally, previous analyses identified that in rice, but not in other graminaceous plants, Fe deficiency resulted in decreased root activity of glutathione reductase, an enzyme required for the ascorbic acid-glutathione cycle to efficiently mediate oxidative stress (Bashir et al. 2007). Accordingly, the rice Fe deficiency response includes accumulation of non-Fe-dependant antioxidants to scavenge ROS and minimise ROS associated damage.

Nitric oxide (NO) is a highly reactive radical that acts as a chemical signal (Gechev et al. 2006) in multiple plant stress responses including; drought, salt, metal toxicity (Spinelli et al. 2011) and Fe deficiency (Arnaud et al. 2006; Vigani et al. 2013). However, NO may also act as an antioxidant to quench superoxide anion radicals (Arasimowicz and Floryszak-Wieczorek 2007). In this study, Fe deficiency was associated with induced levels of citrulline and arginine (Fig. 5e). These results indicate that the Cit-NO cycle was induced which in turn suggests that NO production was increased (Mori 2007). Treatment of *Arabidopsis* with putrescine has previously been shown to alleviate Fe deficiency via the accumulation of NO (Zhu et al. 2016). Similarly, treatment with NO directly has been demonstrated to mitigate the chlorosis associated with Fe deficiency in rice (Lee et al. 2009) and maize (Sun et al. 2007) by acting as an antioxidant. Accordingly, increased NO production may occur as part of the rice Fe deficiency response to signal induction of gene expression and/or mitigate damage associated with oxidative stress.

## Conclusion

The Strategy II Fe deficiency response of rice has been more difficult to study than that of wheat or barley due to very low levels of DMA secretion. The development of a highly sensitive method for the quantification of DMA and NA in this study allowed for the quantification of DMA in rice root secretions which for many previously published methodologies is below the limit of detection. This study reported a thorough characterization of the rice Fe deficiency response. Gene expression and metabolite profiling identified that Fe deficiency induced DMA biosynthesis and DMA secretions followed a diurnal pattern with a peak of induction occurring during 11:00–13:00. The identification of this peak time period, equating to 3–5 h after the onset of light, verified that the peak time of the rice Fe deficiency response is similar to that of other Strategy II plant species such as barley and wheat. However, the diurnal variation in DMA secretion observed within the rice Fe deficiency response was less prominent than that of other graminaceous species described in previous studies. The results presented herein indicate that the mechanisms utilized by rice in response to Fe deficiency include increased DMA-mediated Fe uptake and accumulation of metabolites associated with plant oxidative stress responses. These results constitute an important progression in our understanding of the rice Fe deficiency response.

## Methods

### Plant Materials and Growth Conditions

Rice (*Oryza sativa* L. cv. Nipponbare) grains were surface sterilized with 70% ethyl alcohol and 5% sodium hypochlorite and then germinated on filter paper wetted with 7 mL of reverse osmosis (RO) water in a controlled environment chamber with 12-h photoperiod at 24 °C. After 10 days, germinated rice seedlings were transferred to 13 L supported hydroponic tanks. The rice seedlings were grown for six weeks at a density of thirty plants per hydroponic tank in a growth room with 12-h photoperiod, and 28 °C/24 °C day/night temperature (8:00–20:00/20:00–8:00), with nutrient solution as described in Plett et al. (2010). Rice plants were initially acclimated for one week by growth in ¼ strength Fe-replete growth solution followed by ½ strength Fe-replete growth solution for an additional four days (Additional file 5: Table S3). Plants were grown for a further 4 weeks in full strength Fe-replete growth solution. Growth solutions were replaced every 3–4 days to maintain pH at 5.5. After the four week growth period, Fe-sufficient control plants were grown for an additional week in full strength Fe-replete growth solution while Fe-deficient treatment plants were grown in growth solution lacking NaFe^3+^EDTA.

### Measurement of Plant Size and Chlorophyll Score

Whole plant fresh weight was measured after one week of Fe deficiency treatment. Soil Plant Analysis Development (SPAD) measurements were taken on the youngest leaf (YL) and 2^nd^ youngest leaf (2^nd^ YL) at day 0 and 7 of the Fe deficiency treatment using a Minolta SPAD-502Plus (Spectrum Technologies Inc., Plainfield, Illinois, USA) as an indicator of leaf chlorophyll content.

### Tissue Sampling

Root tissue and root secretions were collected for gene expression and metabolite analyses during the time periods 6:00–8:00 (2–0 h prior to the onset of light), 11:00–13:00 (3–5 h after the onset of light), 18:00–20:00 (10–12 h after the onset of light) and 22:00–24:00 (2–4 h after the change to dark), after one week of Fe deficiency treatment.

### Root Gene Expression Analyses

Extraction of RNA was performed from frozen tissue using the Qiagen RNeasy Mini Kit (Qiagen, Valencia, California, USA) according to manufacturer’s instructions with the exception that 8 μL of the reducing agent dithiothreitol was substituted for 3.5 μL of β-mercaptoethanol (Sigma-Aldrich). Purified RNA was reverse transcribed using the iScript Select cDNA Synthesis Kit (Bio-Rad, Richmond, California, USA) according to manufacturer’s instructions. The expression of *OsNAS1* (LOC_Os03g1942), *OsNAS2* (LOC_Os03g19420), *OsNAS3* (LOC_Os07g48980), *OsNAAT1* (LOC_Os02g20360), *OsDMAS1* (LOC_Os03g13390), *OsTOM1* (LOC_Os11g04020), and *OsYSL15* (LOC_Os02g43410) genes were analysed by quantitative real time PCR (qRT-PCR). The 30 μL reactions contained 6 μL MyTaq Reaction Buffer (Bioline), 7.5 pmol of each primer (Additional file 5: Table S4), 0.15 μL MyTaq polymerase, 25 ng of cDNA and 21.35 μL deionized MilliQ water (dH_2_O). Reactants were initially denatured at 95 °C for 3 min, followed by 35 cycles of 95 °C for 30 s, 55 °C for 30 s, 72 °C for 1 min, and a final extension of 72 °C for 5 min. Expression analyses were performed relative to a three gene normalization factor (3GNF) from expression of the three housekeeping genes: *OsActin* (LOC_Os03g50885), *OsGAPDH* (LOC_Os02g38920), and *OsELF1* (LOC_Os03g29260).

### Root Metabolite Extraction

Frozen root tissue was freeze dried using a Labconco Lyph-lock 6 freeze-dry system (Labconco Corp., Kansas City, Missouri, USA), transferred into 2 mL cryo-mill tubes (Lysing Matrix tube, MP Biomedicals, Solon, Ohio, USA), then 200 μL of methanol (MeOH) was added (containing the internal standards: ^13^C-Sorbitol at 20 μg/mL, ^13^C_5_-^15^N Valine at 20 μg/mL, 2-aminobutyric acid at 10 μg/mL and pentafluorobenzoic acid at 10 μg/mL). The root tissue was then homogenized using a Precellys 24 cryo-mill coupled to a Cryolys (Bertin Technologies, Villeurbanne, France) using the following setting: 3 cycles involving 30 s of milling at 6,800 rpm followed by a 30 s rest period between cycles. The MeOH supernatant was removed and 200 μL of deionized MilliQ water was then added to the cryo-mill tube containing the homogenized root tissue pellet and vortexed for 30 s. The water supernatant was combined with the removed MeOH supernatant and 100 μL of dichloromethane (DCM) was added to the MeOH-water supernatant mixture.

### Root Secretion Collection, Purification and Concentration

Root secretions were collected using the method described in Astolfi et al. (2012) with the following modifications: roots from three plants were briefly rinsed with RO water, transferred into 400 mL of autoclaved dH_2_O in 1 L plastic containers, and then wrapped with aluminium foil to prevent exposure to light. After two hours of root secretion collection, 40 μL of Micropur Forte (Katadyn Products Inc.) was immediately added and filtered through Whatman filter discs. Root secretions were purified using cation exchange Amberlite IR120 H resin (Rohm and Haas Company, Philadelphia, Pennsylvania, USA) with a bed volume (BV) of 50 mL. Sample solutions were eluted through the column at two BV per hour, and this eluate was discarded. Recovery of exchangeable cations (including DMA) was performed by eluting 100 mL of 2 M ammonium hydroxide solution through the Amberlite resin at two BV per hour and stored at −80 °C. Concentration of root secretions was performed through centrifugal evaporation using a RVC 2–25 (Martin Christ Corporation, Osterode, Germany) at 30 °C.

### LC-MS Quantification of Amines

Quantification of root metabolite concentrations was performed as described in Boughton et al. (2011) using an LC 1200 series binary pump with autosampler and heated column compartment coupled to a 6410 series Triple Quadrupole MS (Agilent Technologies Inc.). In brief, derivatization of samples involved combining 10 μL of sample, 70 μL of 200 mM borate buffer (containing the internal standard ^13^C_5_-^15^N-Valine), and 20 μL of fresh 2.85 mg/mL 6-aminoquinolyl-N-hydroxysuccinimidyl carbamate (Aqc) solution. The solution was briefly vortexed, centrifuged at 13,000 rpm for 1 min at 4 °C, then incubated in an Eppendorf thermomixer compact (Eppendorf AG) at 1,150 rpm for 10 min at 55 °C. Reverse phase (RP) chromatography was performed using a Zorbax Eclipse XDB-C18 Rapid Resolution HS 2.1x100 mm 1.8 Micron column (Agilent Technologies Inc.) with a flow rate of 300 μL/min and a temperature of 30 °C. The aqueous mobile phase, solvent ‘A’, was prepared as a 0.1% (v/v) formic acid (FA) in dH_2_O, and the organic mobile phase, solvent ‘B’, was prepared as 0.1% (v/v) FA in acetonitrile. Chromatography was performed using the following gradient:

**Table:** 

Time (min)	A [%]	B [%]	Flow rate (μL/min)
0.00	99	1	300
2.00	99	1	300
9.00	85	15	300
14.00	70	30	300
14.10	99	1	300
19.00	99	1	300

During MS analyses, all metabolites were quantified based on formation of the m/z = 171 Amq product ion. Metabolites were quantified by multiple reaction monitoring (MRM) according to previously published transitions (Boughton et al. 2011; Callahan et al. 2007).

### LC-MS Quantification of DMA and NA

Quantification of 9-fluorenyl methoxycarboxyl chloride (FMOC-Cl) derivatized DMA and NA by RP LC-MS was performed on an LC 1290 series infinity pump with autosampler and heated column compartment coupled to a 6490 series Triple Quadrupole MS (Agilent Technologies Inc.) using a modified version of the method described in Kakei et al. (2009). In short, derivatization of samples involved combining, 5 μL of sample, 10 μL of 1 M sodium borate buffer (pH = 8) containing 42 ng/mL of the internal standard 2-aminoanthracene (2-AA), 10 μL of 50 mM EDTA (pH = 8), and 40 μL of fresh 50 mM FMOC-Cl solution. The solution mixture was incubated at 60 °C with constant shaking at 700 rpm, for 15 min then the reaction was quenched via the addition of 8.9 μL of 5% FA solution (pH = 4). A Zorbax Eclipse XDB-C18 Rapid Resolution HS 2.1x100 mm 1.8 Micron column (Agilent Technologies Inc.) was used during chromatography. The aqueous mobile phase, solvent ‘A’, was prepared as a 0.1% (v/v) FA in dH_2_O, and the organic mobile phase, solvent ‘B’, was prepared as 0.1% (v/v) FA in acetonitrile. Chromatography was performed using the following gradient:

**Table:** 

Time (min)	A [%]	B [%]	Flow rate (μL/min)
0.00	98	2	500
0.50	98	2	500
1.00	40	60	500
4.00	15	85	500
4.10	0	100	500
7.00	0	100	500
7.10	98	2	500
10.00	98	2	500

DMA and NA were observed as [M+(2 × FMOC) + H]^+^ at retention times (RT) 2.88 min and 2.75 min, respectively. Additionally, 2-AA was observed as [M+(FMOC) + H]^+^ at RT 5 min. Quantification of DMA, NA, and 2-AA by MRM used the following transitions:

**Table:** 

	Transition	Precursor (*m/z*)	Product (*m/z*)	Collision energy (V)
[DMA+(2 × FMOC) + H]^+^	Quantifier	749.3	185.8	28
	Qualifier	749.3	139.8	40
[NA+(2 × FMOC) + H]^+^	Quantifier	748.3	526.2	28
	Qualifier	748.3	203.1	42
[2-AA+(FMOC) + H] ^+^	Quantifier	416.2	193.8	34
	Qualifier	416.2	237.7	30

Instrument parameters were set to: high pressure RF 210 V, low pressure RF 150 V, sheath gas temperature 400 °C, sheath gas flow 12 L/min, gas temperature 290 °C, gas flow 16 L/min, nebulizer 20 psi, capillary 4500 V, nozzle voltage 2000 V, cell accelerator voltage 2 V, and delta EMV 250 V.

### Data Processing and Statistical Analyses

Spectra gathered during LC-MS analyses were initially processed using the MassHunter Workstation software package vB.06 (Agilent Technologies Inc.). For LC-MS analyses, compounds were identified by retention time and MRM match to an authentic standard, integrated peak areas were used for quantification relative to external calibration curves. General linear model and Tukey’s test 95% confidence grouping analyses were performed in Minitab 16 statistical software (Minitab Inc., State College, Pennsylvania, USA). Pair-wise statistical analyses were performed using Student’s t-tests between sets of biological replicates from different treatments collected during the same time period only. The metabolomics data analysis server Metaboanalyst 3.0 (Wishart Research Group, the University of Alberta, Edmonton, Alberta, Canada) was used to identify clustering of metabolites profiles between samples based on PCA. Autoscaling and cube-root normalization was performed on analyte levels prior to Metaboanalyst analyses. Permutational ANOVA (PERMANOVA) was used to test for significant differences (*p* < 0.05) in metabolite profiles between Fe treatments and collection times. PERMANOVA tests were run using unrestricted permutation of data and 999 permutations on a resemblance matrix of autoscaled metabolite profiles based on Euclidean distances (Anderson 2005). PERMANOVA tests were performed using PRIMER software (v.6.0) with the PERMANOVA+ add-on (v.1.0.6) (PRIMER-E, Plymouth Marine Laboratory, UK). The Visualization and Analysis of NeTworks with related Experimental Data program, VANTED v2.1.0 was used to map the log base 2 transformations of Fe deficiency associated induction factors of metabolite data on to custom made biochemical pathways maps developed from pathway maps available at the KEGG pathway database (available at http://www.genome.jp/kegg/pathway.html).

## Additional files

Additional file 1: Table S1A. Iron deficiency induced gene expression detected by qRT-PCR. **Table S1B**. Diurnal pattern of Fe deficiency induced gene expression detected by qRT-PCR. (DOCX 21 kb)

Additional file 2: Table S2A. Amines concentrations from Fe-sufficient and Fe-deficient rice roots. **Table S2B**. Iron deficiency associated induction of root amines concentrations. (DOCX 38 kb)

Additional file 3: Figure S1. Levels of 3-P-glycerate in Fe-sufficient and Fe-deficient rice roots quantified by LC-qTOF-MS. **Figure S2**. Levels of ascorbic acid in Fe-sufficient and Fe-deficient rice roots quantified by LC-qTOF-MS. (DOCX 117 kb)

Additional file 4: Figure S3. Induction of precursors to methionine and DMA biosynthesis within the rice Fe deficiency response. (DOCX 831 kb)

Additional file 5: Table S3. Iron replete hydroponic growth solution. **Table S4**. Primers used for qRT-PCR analyses. (DOCX 14 kb)

## Abbreviations

2-AA: 2-aminoanthracene
2^nd^ YL: 2^nd^ youngest leaf
3GNF: 3 gene normalization factor
Aqc: 6-aminoquinolyl-N-hydroxysuccinimidyl carbamate
BV: Bed volume
Cit-NO cycle: Citrulline-nitric oxide cycle
CT: collection times
dH_2_O: deionized MilliQ water
DMA: 2′-deoxymugineic acid
DMAS: 2′-deoxymugineic acid synthase
epiHMA: 3-epihydroxymugineic acid
FA: Formic acid
Fe: Iron
FMOC-Cl: 9-fluorenyl methoxycarboxyl chloride
GOI: Genes of interest
H^+^: proton
IRT: Iron-regulated transporters
LC-MS: Liquid chromatography mass spectrometry
MA: Mugineic acids
MeOH: Methanol
MRM: Multiple reaction monitoring
NA: Nicotianamine
NAAT: Nicotianamine aminotransferase
NAS: Nicotianamine synthase
NO: Nitric oxide
PCA: Principal component analysis
PERMANOVA: Permutational ANOVA
ppm: parts per million
PS: Phytosiderophores
qRT-PCR: Quantitative real time PCR
RO water: Reverse osmosis water
ROS: Reactive oxygen species
RP chromatography: Reverse phase chromatography
RT: Retention times
SAM: S-adenosyl-methionine
SE: Standard error
SPAD: Soil plant analysis development
TOM: Transporter of mugineic acid family phytosiderophores
Tukey’s HSD test: Tukey’s honest significant difference test
VANTED: Visualization and analysis of networks with related experimental data program
YL: Youngest leaf
YSL: Yellow stripe-like

## References

[CR1] Anderson MJ (2005) Permutational multivariate analysis of variance. Department of Statistics, University of Auckland, Auckland 26:32–46.

[CR2] Arasimowicz M, Floryszak-Wieczorek J (2007). Nitric oxide as a bioactive signalling molecule in plant stress responses. Plant Sci.

[CR3] Ariga T, Hazama K, Yanagisawa S, Yoneyama T (2014). Chemical forms of iron in xylem sap from graminaceous and non-graminaceous plants. Soil Sci Plant Nutr.

[CR4] Arnaud N, Murgia I, Boucherez J, Briat J-F, Cellier F, Gaymard F (2006). An iron-induced nitric oxide burst precedes ubiquitin-dependent protein degradation for *Arabidopsis AtFer1* ferritin gene expression. J Biol Chem.

[CR5] Astolfi S, Zuchi S, Neumann G, Cesco S, Di Toppi LS, Pinton R (2012). Response of barley plants to Fe deficiency and Cd contamination as affected by S starvation. J Exp Bot.

[CR6] Bashir K, Nagasaka S, Itai RN, Kobayashi T, Takahashi M, Nakanishi H, Mori S, Nishizawa NK (2007). Expression and enzyme activity of glutathione reductase is upregulated by Fe-deficiency in graminaceous plants. Plant Mol Biol.

[CR7] Becana M, Moran JF, Iturbe-Ormaetxe I, Escuredo PR (1998). Iron-dependent oxygen free radical generation in plants subjected to environmental stress: toxicity and antioxidant protection. Plant Soil.

[CR8] Bhullar NK, Gruissem W (2013). Nutritional enhancement of rice for human health: the contribution of biotechnology. Biotechnol Adv.

[CR9] Boughton BA, Callahan DL, Silva C, Bowne J, Nahid A, Rupasinghe T, Tull DL, McConville MJ, Bacic A, Roessner U (2011). Comprehensive profiling and quantitation of amine group containing metabolites. Anal Chem.

[CR10] Briat J-F, Dubos C, Gaymard F (2015). Iron nutrition, biomass production, and plant product quality. Trends Plant Sci.

[CR11] Bughio N, Yamaguchi H, Nishizawa NK, Nakanishi H, Mori S (2002). Cloning an iron-regulated metal transporter from rice. J Exp Bot.

[CR12] Callahan DL, Kolev SD, O’Hair RAJ, Salt DE, Baker AJM (2007). Relationships of nicotianamine and other amino acids with nickel, zinc and iron in *Thlaspi* hyperaccumulators. New Phytol.

[CR13] Chen L, Ding C, Zhao X, Xu J, Mohammad AA, Wang S, Ding Y (2015). Differential regulation of proteins in rice (*Oryza sativa* L.) under iron deficiency. Plant Cell Rep.

[CR14] Clemens S (2001). Molecular mechanisms of plant metal tolerance and homeostasis. Planta.

[CR15] Connolly EL, Guerinot ML (2002). Iron stress in plants. Genome Biol.

[CR16] da Silveira VC, de Oliveira AP, Sperotto RA, Espindola LS, Amaral L, Dias JF, da Cunha JB, Fett JP (2007). Influence of iron on mineral status of two rice (*Oryza sativa* L.) cultivars. Braz J Plant Physiol.

[CR17] Dell’mour M, Koellensperger G, Quirino JP, Haddad PR, Stanetty C, Oburger E, Puschenreiter M, Hann S (2010). Complexation of metals by phytosiderophores revealed by CE-ESI-MS and CE-ICP-MS. Electrophoresis.

[CR18] Di Martino C, Delfine S, Pizzuto R, Loreto F, Fuggi A (2003). Free amino acids and glycine betaine in leaf osmoregulation of spinach responding to increasing salt stress. New Phytol.

[CR19] Droux M, Ravanel S, Douce R (1995). Methionine biosynthesis in higher plants. II. Purification and characterization of cystathionine *β*-lyase from spinach chloroplasts. Arch Biochem Biophys.

[CR20] Ercal N, Gurer-Orhan H, Aykin-Burns N (2001). Toxic metals and oxidative stress part I: mechanisms involved in metal-induced oxidative damage. Curr Top Med Chem.

[CR21] Fan TW-M, Lane AN, Shenker M, Bartley JP, Crowley D, Higashi RM (2001). Comperhensive chemical profiling of gramineous plant root exudates using high-resolution NMR and MS. Phytochemistry.

[CR22] Gechev TS, Breusegem FV, Stone JM, Denev I, Laloi C (2006). Reactive oxygen species as signals that modulate plant stress responses and programmed cell death. BioEssays.

[CR23] Ghasemzadeh A, Ghasemzadeh N (2011). Flavonoids and phenolic acids: role and biochemical activity in plants and human. J Med Plants Res.

[CR24] Gill SS, Tuteja N (2010). Reactive oxygen species and antioxidant machinery in abiotic stress tolerance in crop plants. Plant Physiol Biochem.

[CR25] Hell R, Stephan UW (2003). Iron uptake, trafficking and homeostasis in plants. Planta.

[CR26] Hider RC, Yoshimura E, Khodr H, von Wirén N (2004). Competition or complementation: the iron-chelating abilities of nicotianamine and phytosiderophores. New Phytol.

[CR27] Higuchi K, Kanazawa K, Nishizawa N-K, Mori S (1996). The role of nicotianamine synthase in response to Fe nutrition status in Gramineae. Plant Soil.

[CR28] Higuchi K, Watanabe S, Takahashi M, Kawasaki S, Nakanishi H, Nishizawa NK, Mori S (2001). Nicotianamine synthase gene expression differs in barley and rice under Fe-deficienct conditions. Plant J.

[CR29] Hung C-H, Hwang HJ, Chen Y-H, Chiu Y-F, Ke S-C, Burnap RL, Chu H-A (2010). Spectroscopic and functional characterizations of cyanobacterium *Synechocystis* PCC 6803 mutants on and near the heme axial ligand of cytochrome b559 in photosystem II. J Biol Chem.

[CR30] Inoue H, Higuchi K, Takahashi M, Nakanishi H, Mori S, Nishizawa NK (2003). Three rice nicotianamine synthase genes, *OsNAS1*, *OsNAS2*, and *OsNAS3* are expressed in cells involved in long-distance transport of iron and differentially regulated by iron. Plant J.

[CR31] Inoue H, Kobayashi T, Nozoye T, Takahashi M, Kakei Y, Suzuki K, Nakazono M, Nakanishi H, Mori S, Nishizawa NK (2009). Rice OsYSL15 is an iron-regulated iron(III)-deoxymugineic acid transporter expressed in the roots and is essential for iron uptake in early growth of the seedlings. J Biol Chem.

[CR32] Ishimaru Y, Suzuki M, Tsukamoto T, Suzuki K, Nakazono M, Kobayashi T, Wada Y, Watanabe S, Matsuhashi S, Takahashi M, Nakanishi H, Mori S, Nishizawa NK (2006). Rice plants take up iron as an Fe^3+^ phytosiderophore and as Fe^2+^. Plant J.

[CR33] Itai RN, Ogo Y, Kobayashi T, Nakanishi H, Nishizawa NK (2013). Rice genes involved in phytosiderophore biosynthesis are synchronously regulated during the early stages of iron deficiency in roots. Rice.

[CR34] Iturbe-Ormaetxe I, Moran JF, Arrese-Igor C, Gogorcena Y, Klucas RV, Becana M (1995). Activated oxygen and antioxidant defenses in iron-deficient pea plants. Plant Cell Environ.

[CR35] Joshi V, Joung J-G, Fei Z, Jander G (2010). Interdependence of threonine, methionine and isoleucine metabolism in plants: accumulation and transcriptional regulation under abiotic stress. Amino Acids.

[CR36] Kakei Y, Yamaguchi I, Kobayashi T, Takahashi M, Nakanishi H, Yamakawa T, Nishizawa NK (2009). A highly sensitive, quick and simple quantification method for nicotianamine and 2′-deoxymugineic acid from minimum samples using LC/ESI-TOF-MS achieves functional analysis of these components in plants. Plant Cell Physiol.

[CR37] Kawai S, Itoh K, Takagi SI (1993). Incorporation of ^15^N and ^14^C of methionine into the mugineic acid family of phytosiderophores in iron-deficient barley roots. Physiol Plant.

[CR38] Kim SA, Guerinot ML (2007). Mining iron: iron uptake and transport in plants. FEBS Lett.

[CR39] Kobayashi T, Itai RN, Ogo Y, Kakei Y, Nakanishi H, Takahashi M, Nishizawa NK (2009). The rice transcription factor IDEF1 is essential for the early response to iron deficiency, and induces vegetative expression of late embryogenesis abundant genes. Plant J.

[CR40] Kosová K, Vítámvás P, Prášil IT, Renaut J (2011). Plant proteome changes under abiotic stress - Contribution of proteomics studies to understanding plant stress response. J Proteome.

[CR41] Lee S, An G (2009). Over-expression of OsIRT1 leads to increased iron and zinc accumulations in rice. Plant, Cell and Environment.

[CR42] Lee S, Chiecko JC, Kim SA, Walker EL, Lee Y, Guerinot ML, An G (2009). Disruption of *OsYSL15* leads to iron inefficiency in rice plants. Plant Physiol.

[CR43] Li S, Zhou X, Huang Y, Zhu L, Zhang S, Zhao Y, Guo J, Chen J, Chen R (2013). Identification and characterization of the zinc-regulated transporters, iron-regulated transporter-like protein (ZIP) gene family in maize. BMC Plant Biol.

[CR44] Ma JF, Shinada T, Matsuda C, Nomoto K (1995). Biosynthesis of phytosiderophores, mugineic acids, associated with methionine cycling. J Biol Chem.

[CR45] Ma JF, Ueno H, Ueno D, Rombolà AD, Iwashita T (2003). Characterization of phytosiderophore secretion under Fe deficiency stress in *Festuca rubra*. Plant Soil.

[CR46] Marschner H, Römheld V, Kissel M (1986). Different strategies in higher plants in mobilization and uptake of iron. J Plant Nutr.

[CR47] Marsh HV, Evans HJ, Matrone G (1963). Investigations of the role of iron in chlorophyll metabolism I. Effect of iron deficiency on chlorophyll and heme content and on the activities of certain enzymes in leaves. Plant Physiol.

[CR48] Michalak A (2006). Phenolic compounds and their antioxidant activity in plants growing under heavy metal stress. Pol J Environ Stud.

[CR49] Michel K-P, Pistorius EK (2004). Adaptation of the photosynthetic electron transport chain in cyanobacteria to iron deficiency: the function of IdiA and IsiA. Physiol Plant.

[CR50] Miller GW, Pushnik JC, Welkie GW (1984). Iron chlorosis, a world wide problem, the relation of chlorophyll biosynthesis to iron. J Plant Nutr.

[CR51] Mori M (2007). Regulation of nitric oxide synthesis and apoptosis by arginase and arginine recycling. J Nutr.

[CR52] Mori S, Nishizawa NK (1987). Methionine as a dominant precursor of phytosiderophores in *Graminaceae* plants. Plant Cell Physiol.

[CR53] Mori S, Nishizawa N, Kawai S, Sato Y, Takagi S (1987). Dynamic state of mugineic acid and analogous phytosiderophores in Fe‐deficient barley. J Plant Nutr.

[CR54] Mori S, Nishizawa N, Hayashi H, Chino M, Yoshimura E, Ishihara J (1991). Why are young rice plants highly susceptible to iron deficiency?. Plant Soil.

[CR55] Nagasaka S, Takahashi M, Nakanishi-Itai R, Bashir K, Nakanishi H, Mori S, Nishizawa NK (2009). Time course analysis of gene expression over 24 h in Fe-deficient barley roots. Plant Mol Biol.

[CR56] Nakanishi H, Bughio N, Matsuhashi S, Ishioka N-S, Uchida H, Tsuji A, Osa A, Sekine T, Kume T, Mori S (1999). Visualizing real time [^11^C] methionine translocation in Fe-sufficient and Fe-deficient barley using a Positron Emitting Tracer Imaging System (PETIS). J Exp Bot.

[CR57] Negishi T, Nakanishi H, Yazaki J, Kishimoto N, Fujii F, Shimbo K, Yamamoto K, Sakata K, Sasaki T, Kikuchi S, Mori S, Nishizawa NK (2002). cDNA microarray analysis of gene expression during Fe-deficiency stress in barley suggests that polar transport of vesicles is implicated in phytosiderophore secretion in Fe-deficient barley roots. Plant J.

[CR58] Nimalaratne C, Lopes-Lutz D, Schieber A, Wu J (2011). Free aromatic amino acids in egg yolk show antioxidant properties. Food Chem.

[CR59] Nishiyama H, Ohya T, Tanoi K, Nakanishi TM (2008). A simple measurement of the pH of root apoplast by the fluorescence ratio method. Plant Root.

[CR60] Nishiyama R, Kato M, Nagata S, Yanagisawa S, Yoneyama T (2012). Identification of Zn–Nicotianamine and Fe–2′-Deoxymugineic acid in the phloem sap from rice plants (*Oryza sativa* L.). Plant Cell Physiol.

[CR61] Nozoye T, Itai RN, Nagasaka S, Takahashi M, Nakanishi H, Mori S, Nishizawa NK (2004). Diurnal changes in the expression of genes that participate in phytosiderophore synthesis in rice. Soil Sci Plant Nutr.

[CR62] Nozoye T, Nagasaka S, Kobayashi T, Takahashi M, Sato Y, Sato Y, Nakanishi NUH, Nishizawa NK (2011). Phytosiderophore efflux transporters are crucial for iron acquisition in graminaceous plants. J Biol Chem.

[CR63] Nozoye T, Nagasaka S, Bashir K, Takahashi M, Kobayashi T, Nakanishi H, Nishizawa NK (2014). Nicotianamine synthase 2 localizes to the vesicles of iron-deficient rice roots, and its mutation in the YXXφ or LL motif causes the disruption of vesicle formation or movement in rice. Plant J.

[CR64] Oburger E, Gruber B, Schindlegger Y, Schenkeveld WDC, Hann S, Kraemer SM, Wenzel WW, Puschenreiter M (2014). Root exudation of phytosiderophores from soil-grown wheat. New Phytol.

[CR65] Parida AK, Das AB (2005). Salt tolerance and salinity effects on plants: a review. Ecotoxicol Environ Saf.

[CR66] Park S, Pan L-P, Chan SI, Alben JO (1996). Photoperturbation of the heme a_3_-Cu_B_ binuclear center of cytochrome c oxidase CO complex observed by Fourier transform infrared spectroscopy. Biophys J.

[CR67] Pedas P, Ytting CK, Fuglsang AT, Jahn TP, Schjoerring JK, Husted S (2008). Manganese efficiency in barley: identification and characterization of the metal ion transporter HvIRT1. Plant Physiol.

[CR68] Plett D, Safwat G, Gilliham M, Møller IS, Roy S, Shirley N, Jacobs A, Johnson A, Tester M (2010). Improved salinity tolerance of rice through cell type-specific expression of *AtHKT1;1*. PLoS ONE.

[CR69] Rai VK (2002). Role of amino acids in plant responses to stresses. Biol Plant.

[CR70] Ramakrishna A, Ravishankar GA (2011). Influence of abiotic stress signals on secondary metabolites in plants. Plant Signal Behav.

[CR71] Reddy KJ, Rao KVM, Raghavendra AS, Reddy KJ (2006). Nutrient Stress. Physiology and molecular biology of stress tolerance in plants.

[CR72] Ricachenevsky FK, Sperotto RA (2014). There and back again, or always there? The evolution of rice combined strategy for Fe uptake. Front Plant Sci.

[CR73] Römheld V, Marschner H (1990). Genotypical differences among graminaceous species in release of phytosiderophores and uptake of iron phytosiderophores. Plant Soil.

[CR74] Rong-li S, Hong-mei H, Xiao-yun F, Karim MR, Fu-suo Z, Chun-qin Z (2012). Responses of aerobic rice (*Oryza sativa* L.) to iron deficiency. J Integr Agric.

[CR75] Scholz G, Becker R, Pich A, Stephan UW (1992). Nicotianamine - a common constituent of strategies I and II of iron acquisition by plants: a review. J Plant Nutr.

[CR76] Sharma SS, Dietz K-J (2006). The significance of amino acids and amino acid-derived molecules in plant responses and adaptation to heavy metal stress. J Exp Bot.

[CR77] Sperotto RA, Ricachenevsky FK, De Abreu Waldow V, Fett JP (2012). Iron biofortification in rice: it’s a long way to the top. Plant Sci.

[CR78] Spinelli F, Cellini A, Marchetti L, Nagesh KM, Piovene C, Shanker A, Venkateswarlu B (2011). Emission and function of volatile organic compounds in response to abiotic stress. Abiotic stress in plants - mechanisms and adaptations.

[CR79] Stadtman ER, Levine RL (2003). Free radical-mediated oxidation of free amino acids and amino acid residues in proteins. Amino Acids.

[CR80] Stocking CR (1975). Iron deficiency and the structure and physiology of maize chloroplasts. Plant Physiol.

[CR81] Sun B, Jing Y, Chen K, Song L, Chen F, Zhang L (2007). Protective effect of nitric oxide on iron deficiency-induced oxidative stress in maize (*Zea mays*). J Plant Physiol.

[CR82] Suzuki M, Takahashi M, Tsukamoto T, Watanabe S, Matsuhashi S, Yazaki J, Kishimoto N, Kikuchi S, Nakanishi H, Mori S, Nishizawa NK (2006). Biosynthesis and secretion of mugineic acid family phytosiderophores in zinc-deficient barley. Plant J.

[CR83] Takagi SI, Nomoto K, Takemoto T (1984). Physiological aspect of mugineic acid, a possible phytosiderophore of graminaceous plants. J Plant Nutr.

[CR84] Terry N, Low G (1982). Leaf chlorophyll content and its relation to the intracellular localization of iron. J Plant Nutr.

[CR85] Ueno D, Yamaji N, Ma JF (2009). Further characterization of ferric—phytosiderophore transporters ZmYS1 and HvYS1 in maize and barley. J Exp Bot.

[CR86] Vasconcelos M, Musetti V, Li C-M, Datta SK, Grusak MA (2004). Functional analysis of transgenic rice (*Oryza sativa* L.) transformed with an *Arabidopsis thaliana* ferric reductase (AtFR02). Soil Sci Plant Nutr.

[CR87] Vigani G, Zocchi G, Bashir K, Philippar K, Briat J-F (2013). Signals from chloroplasts and mitochondria for iron homeostasis regulation. Trends Plant Sci.

[CR88] Walker EL, Connolly EL (2008). Time to pump iron: iron-deficiency-signaling mechanisms of higher plants. Curr Opin Plant Biol.

[CR89] Walter A, Pich A, Scholz G, Marschner H, Römheld V (1995). Diurnal variations in release of phytosiderophores and in concentrations of phytosiderophores and nicotianamine in roots and shoots of barley. J Plant Physiol.

[CR90] Wang L, Ying Y, Narsai R, Ye L, Zheng L, Tian J, Whelan J, Shou H (2013). Identification of OsbHLH133 as a regulator of iron distribution between roots and shoots in *Oryza sativa*. Plant Cell Environ.

[CR91] Weber G, von Wirén N, Hayen H (2008). Investigation of ascorbate-mediated iron release from ferric phytosiderophores in the presence of nicotianamine. BioMetals.

[CR92] Wingler A, Lea PJ, Quick WP, Leegood RC (2000). Photorespiration: metabolic pathways and their role in stress protection. Philos Trans R Soc Lond B Biol Sci.

[CR93] Yoneyama T, Ishikawa S, Fujimaki S (2015). Route and regulation of Zinc, Cadmium, and Iron transport in rice plants (*Oryza sativa* L.) during vegetative growth and grain filling: Metal transporters, metal speciation, grain Cd reduction and Zn and Fe biofortification. Int J Mol Sci.

[CR94] Zaharieva TB, Abadía J (2003). Iron deficiency enhances the levels of ascorbate, glutathione, and related enzymes in sugar beet roots. Protoplasma.

[CR95] Zhang F-S, Römheld V, Marschner H (1991). Diurnal rhythm of release of phytosiderophores and uptake rate of zinc in iron-deficient wheat. Soil Sci Plant Nutr.

[CR96] Zhang X, Zhang F, Mao D (1998). Effect of iron plaque outside roots on nutrient uptake by rice (*Oryza sativa* L.). Zinc uptake by Fe-deficient rice. Plant Soil.

[CR97] Zhu XF, Wang B, Song WF, Zheng SJ, Shen RF (2016). Putrescine alleviates iron deficiency via NO-dependent reutilization of root cell-wall Fe in Arabidopsis. Plant Physiol.

[CR98] Zoghlami LB, Djebali W, Abbes Z, Hediji H, Maucourt M, Moing A, Brouquisse R, Chaïbi W (2011). Metabolite modifications in *Solanum lycopersicum* roots and leaves under cadmium stress. Afr J Biotechnol.

