# Supplementary material for: Diurnal Changes in Transcript and Metabolite Levels during the Iron Deficiency Response of Rice
Source: Rice (N Y). 2017 Apr 20;10:14. doi: 10.1186/s12284-017-0152-7 (PMC5398970; doi:10.1186/s12284-017-0152-7)
Supplement: Supplementary file 1 — Iron deficiency induced gene expression detected by qRT-PCR. Table S1B. Diurnal pattern of Fe deficiency induced gene expression detected by qRT-PCR. (DOCX 21 kb) [file 12284_2017_152_MOESM1_ESM.docx]

# Table S1A Iron deficiency induced gene expression detected by qRT-PCR. Values presented are *p*-values from Student’s t-tests and One-way ANOVA. Significant differences are indicated by * for *p* < 0.05, ** for *p* < 0.01 and *** for *p* ≤ 0.001 for Tukey’s honest significant difference (HSD) tests performed between treatments across all collection times (CT) and Student’s t-tests performed between treatments within collection times.

| Gene | *p*-values | | | | | | |
| --- | --- | --- | --- | --- | --- | --- | --- |
|  | Between treatments across CT | +Fe  across CT | -Fe  across CT | 6:00 CT | 11:00 CT | 18:00 CT | 22:00 CT |
| *OsNAS1* | ≤ 0.001 *** | 0.685 | 0.016 * | 0.029 * | 0.003 ** | 0.139 | 0.203 |
| *OsNAS2* | ≤ 0.001 *** | 0.668 | 0.017 * | 0.024 * | 0.004 ** | 0.097 | 0.257 |
| *OsNAS3* | ≤ 0.001 *** | 0.012 * | 0.124 | ≤ 0.001 *** | 0.059 | 0.172 | 0.111 |
| *OsNAAT1* | ≤ 0.001 *** | 0.590 | ≤ 0.001 *** | 0.038 * | ≤ 0.001 *** | 0.414 | 0.133 |
| *OsDMAS1* | ≤ 0.001 *** | 0.241 | 0.049 * | 0.056 | 0.005 ** | 0.217 | 0.034 * |
| *OsTOM1* | 0.054 | 0.716 | 0.012 * | 0.031 * | 0.066 | 0.725 | 0.388 |
| *OsYSL15* | 0.006 ** | 0.064 | 0.746 | 0.046 * | 0.014 * | 0.196 | 0.850 |

# Table S1B Diurnal pattern of Fe deficiency induced gene expression detected by qRT-PCR. Values presented are means ± SE of 9 biological replicates of the expression of the genes of interest (GOI) relative to the 3 gene normalization factor (3GNF). Iron deficiency associated induction factor (IF) is defined as the Fe-deficient (-Fe) mean expression divided by the Fe-sufficient (+Fe) mean expression. Significant differences are indicated by * for *p* < 0.05, ** for *p* < 0.01, *** for *p* ≤ 0.001 by Student’s t-tests performed between treatments within collection times.

| Collection time | Treatment | *OsNAS1* | *OsNAS2* | *OsNAS3* | *OsNAAT1* | *OsDMAS1* | *OsTOM1* | *OsYSL15* |
| --- | --- | --- | --- | --- | --- | --- | --- | --- |
| 6:00 | +Fe | 59581 ± 10377 | 60662 ± 9456 | 320.9 ± 29.8 | 40331 ± 5510 | 17177 ± 2470 | 4715 ± 1062 | 6895 ± 1076 |
|  | -Fe | 121375 ± 23671 | 114306 ± 19239 | 1115 ± 173 | 61344 ± 9204 | 32097 ± 6907 | 6855 ± 698 | 12453 ± 2531 |
|  | IF | 2.04* | 1.88* | 3.47*** | 1.52* | 1.87 | 1.45* | 1.81* |
| 11:00 | +Fe | 47868 ± 11644 | 47808 ± 12211 | 441 ± 144 | 30143 ± 6439 | 11526 ± 2108 | 5512 ± 1546 | 6126 ± 1882 |
|  | -Fe | 137495 ± 20523 | 138300 ± 21469 | 928 ± 146 | 75230 ± 7700 | 12566 ± 5311 | 12566 ± 3036 | 14724 ± 2521 |
|  | IF | 2.87** | 2.89** | 2.10 | 2.50*** | 2.79** | 2.28 | 2.40* |
| 18:00 | +Fe | 45694 ± 12950 | 47833 ± 12603 | 643 ± 111 | 29931 ± 7950 | 12634 ± 2697 | 6237 ± 2546 | 11609 ± 1636 |
|  | -Fe | 75026 ± 10293 | 81078 ± 10725 | 972 ± 210 | 38694 ± 4782 | 17828 ± 2409 | 5196 ± 1167 | 16435 ± 2978 |
|  | IF | 1.64 | 1.70 | 1.51 | 1.29 | 1.41 | 0.83 | 1.42 |
| 22:00 | +Fe | 41895 ± 9148 | 41848 ± 10816 | 885 ± 169 | 30045 ± 5081 | 11536 ± 1822 | 3663 ± 901 | 12676 ± 2946 |
|  | -Fe | 67363 ± 14413 | 64791 ± 10816 | 1667 ± 360 | 44334 ± 5797 | 20391 ± 2719 | 5149 ± 1232 | 13599 ± 3126 |
|  | IF | 1.61 | 1.55 | 1.88 | 1.48 | 1.77* | 1.41 | 1.07 |
