# Supplementary material for: Diurnal Changes in Transcript and Metabolite Levels during the Iron Deficiency Response of Rice
Source: Rice (N Y). 2017 Apr 20;10:14. doi: 10.1186/s12284-017-0152-7 (PMC5398970; doi:10.1186/s12284-017-0152-7)
Supplement: Supplementary file 2 — Amines concentrations from Fe-sufficient and Fe-deficient rice roots. Table S2B. Iron deficiency associated induction of root amines concentrations. (DOCX 38 kb) [file 12284_2017_152_MOESM2_ESM.docx]

# Table S2A Amines concentrations from Fe-sufficient and Fe-deficient rice roots. Values presented are means (nmol /g DW) ± SE of 5 biological replicates. Iron deficiency associated induction factor (IF) is defined as the Fe-deficient (-Fe) mean concentration divided by the Fe-sufficient (+Fe) mean concentrations. Significant differences are indicated by * for *p* < 0.05, ** for *p* < 0.01, *** for *p* ≤ 0.001 by Student’s t-tests performed between treatments within collection times.

| Metabolite | 6:00 Collection time | | |
| --- | --- | --- | --- |
|  | +Fe | -Fe | IF |
| 4-hydroxy-proline | 27.49 ± 1.79 | 26.75 ± 1.53 | 0.97 |
| Histidine | 814.09 ± 25.08 | 951.93 ± 56.74 | 1.17 |
| Asparagine | 9174.75 ± 1053.59 | 7730.34 ± 812.92 | 0.84 |
| Arginine | 444.59 ± 37.3 | 756.76 ± 105.25 | 1.7* |
| Taurine | 19.46 ± 0.82 | 20.74 ± 1.35 | 1.07 |
| Serine | 3162.84 ± 211.36 | 4454.46 ± 225.03 | 1.41** |
| Glutamine | 27920.02 ± 3972.04 | 29474.96 ± 1564.92 | 1.06 |
| Homoserine | 336.47 ± 18.53 | 333.17 ± 27.83 | 0.99 |
| Glycine | 564.36 ± 43.37 | 679.84 ± 24.41 | 1.2* |
| Aspartate | 35768.74 ± 1977.72 | 32178.12 ± 2132.64 | 0.9 |
| Citrulline | 60.72 ± 4.03 | 75.03 ± 2.54 | 1.24* |
| Glutamate | 27592.19 ± 1525.77 | 27933.38 ± 1629.43 | 1.01 |
| beta-Alanine | 672.2 ± 117.56 | 1205.76 ± 141.05 | 1.79* |
| Threonine | 1199.67 ± 78.78 | 1647.23 ± 85.81 | 1.37** |
| Alanine | 5921.61 ± 863.89 | 6254.95 ± 1035.52 | 1.06 |
| GABA | 3313.23 ± 310.64 | 3658.62 ± 686.84 | 1.1 |
| Proline | 709.18 ± 76.92 | 1300.01 ± 163.63 | 1.83** |
| Cysteine | 347.68 ± 9.62 | 446.22 ± 16.44 | 1.28*** |
| Ornithine | 36.49 ± 2.27 | 57.7 ± 17.08 | 1.58 |
| Octopamine | 84.5 ± 9.62 | 89.26 ± 5.34 | 1.06 |
| Lysine | 383.5 ± 26.26 | 474.33 ± 31.75 | 1.24 |
| Putrescine | 2886.58 ± 126.71 | 2660.35 ± 62.47 | 0.92 |
| Cadaverine | 157.21 ± 25.9 | 157.44 ± 11.35 | 1 |
| Tyrosine | 975.27 ± 41.27 | 1133.62 ± 34.52 | 1.16* |
| Methionine | 105.94 ± 10.08 | 166.96 ± 13.14 | 1.58** |
| Valine | 1284.71 ± 131.81 | 2035.94 ± 117.25 | 1.58** |
| Serotonin | 119.54 ± 14.88 | 236.99 ± 25.74 | 1.98** |
| Tyramine | 492.4 ± 97.64 | 567.63 ± 37.6 | 1.15 |
| Isoleucine | 82.09 ± 11.48 | 190.36 ± 19.02 | 2.32*** |
| Leucine | 498.3 ± 35.53 | 659.8 ± 32.6 | 1.32** |
| Phenylalanine | 543.11 ± 47.17 | 620.99 ± 34.89 | 1.14 |
| Tryptophan | 421.28 ± 10.95 | 599.12 ± 46.49 | 1.42** |
| Nicotianamine | 391.51 ± 76.89 | 259.5 ± 70.37 | 0.66 |
| 2’-Deoxymugineic acid | 7302.07 ± 661.43 | 9013.14 ± 678.78 | 1.23 |

| Metabolite | 11:00 Collection time | | |
| --- | --- | --- | --- |
|  | +Fe | -Fe | IF |
| 4-hydroxy-proline | 26.27 ± 1.5 | 27.87 ± 3.05 | 1.06 |
| Histidine | 750.57 ± 21.35 | 1042.82 ± 70.18 | 1.39** |
| Asparagine | 6585.42 ± 809.74 | 6154.34 ± 774.3 | 0.93 |
| Arginine | 400.21 ± 34.28 | 986.82 ± 66.54 | 2.47*** |
| Taurine | 17.47 ± 0.83 | 21.71 ± 1.52 | 1.24* |
| Serine | 3228.17 ± 137.09 | 4328.93 ± 219.95 | 1.34** |
| Glutamine | 18059.12 ± 834.95 | 22462.34 ± 1364.65 | 1.24* |
| Homoserine | 373.19 ± 45.58 | 323.36 ± 34.5 | 0.87 |
| Glycine | 482.32 ± 43.73 | 625.15 ± 24.1 | 1.3* |
| Aspartate | 29730.16 ± 1817.99 | 26877.95 ± 1758.69 | 0.9 |
| Citrulline | 56.66 ± 1.56 | 67.66 ± 7.22 | 1.19 |
| Glutamate | 23046.19 ± 1821.77 | 24332.14 ± 724.06 | 1.06 |
| beta-Alanine | 388.09 ± 53.09 | 1069.51 ± 109.92 | 2.76*** |
| Threonine | 1170.54 ± 68.63 | 1733.15 ± 110.82 | 1.48** |
| Alanine | 3831.73 ± 379.47 | 4935.43 ± 503.3 | 1.29 |
| GABA | 2862.71 ± 753.88 | 3393.48 ± 276.17 | 1.19 |
| Proline | 438.92 ± 41.55 | 771.21 ± 66.65 | 1.76** |
| Cysteine | 358.54 ± 27.92 | 446.74 ± 21.37 | 1.25* |
| Ornithine | 41.78 ± 8.95 | 49.7 ± 13.22 | 1.19 |
| Octopamine | 88.83 ± 5.41 | 98.32 ± 5.01 | 1.11 |
| Lysine | 431.76 ± 39.28 | 737.64 ± 63.18 | 1.71** |
| Putrescine | 2723.35 ± 203.46 | 2728.48 ± 208.12 | 1 |
| Cadaverine | 142.5 ± 11.22 | 157.64 ± 9.68 | 1.11 |
| Tyrosine | 746.5 ± 57.69 | 1006.09 ± 20.17 | 1.35** |
| Methionine | 102.87 ± 8.02 | 170.2 ± 19.04 | 1.65* |
| Valine | 980.8 ± 49.45 | 2225.24 ± 235.75 | 2.27*** |
| Serotonin | 182.06 ± 22.84 | 319.46 ± 44.02 | 1.75* |
| Tyramine | 658.33 ± 78.33 | 669.58 ± 89.73 | 1.02 |
| Isoleucine | 125.6 ± 14.71 | 246.81 ± 36.1 | 1.96* |
| Leucine | 383.29 ± 20.71 | 776.61 ± 68.65 | 2.03*** |
| Phenylalanine | 417.75 ± 44.56 | 714.04 ± 50.96 | 1.71** |
| Tryptophan | 385.49 ± 18.14 | 680.41 ± 41.1 | 1.77*** |
| Nicotianamine | 206.07 ± 32.21 | 312.91 ± 76.56 | 1.52 |
| 2’-Deoxymugineic acid | 4904.25 ± 397.8 | 7580.51 ± 529.64 | 1.55** |

| Metabolite | 18:00 Collection time | | |
| --- | --- | --- | --- |
|  | +Fe | -Fe | IF |
| 4-hydroxy-proline | 24 ± 2.11 | 26.57 ± 3.09 | 1.11 |
| Histidine | 652.78 ± 58.99 | 979.98 ± 77.92 | 1.5** |
| Asparagine | 4808.12 ± 721.83 | 8725.68 ± 454.99 | 1.81** |
| Arginine | 388.43 ± 70.55 | 596.52 ± 75.99 | 1.54 |
| Taurine | 17.58 ± 1.12 | 21.6 ± 3.1 | 1.23 |
| Serine | 2786.92 ± 234.25 | 4618.17 ± 142.37 | 1.66*** |
| Glutamine | 15726.39 ± 988.69 | 32279.9 ± 1934.26 | 2.05*** |
| Homoserine | 276.27 ± 23.85 | 362.44 ± 56.34 | 1.31 |
| Glycine | 381.12 ± 37.56 | 655.62 ± 37.99 | 1.72*** |
| Aspartate | 24220.12 ± 1932.95 | 29575.27 ± 3045.76 | 1.22 |
| Citrulline | 52.82 ± 3.84 | 73.97 ± 2.51 | 1.4** |
| Glutamate | 22710.49 ± 1970.53 | 27934.9 ± 1767.87 | 1.23 |
| beta-Alanine | 369.49 ± 54.82 | 942.37 ± 95.74 | 2.55*** |
| Threonine | 1089.06 ± 104.25 | 1816.91 ± 91 | 1.67*** |
| Alanine | 3007.46 ± 127.4 | 7274.48 ± 919.3 | 2.42** |
| GABA | 1949.8 ± 220.71 | 2398.21 ± 376.56 | 1.23 |
| Proline | 365.25 ± 33.34 | 1080.05 ± 169.49 | 2.96** |
| Cysteine | 347.64 ± 26.82 | 464.17 ± 65.76 | 1.34 |
| Ornithine | 44.06 ± 6.33 | 47.73 ± 9.25 | 1.08 |
| Octopamine | 89.49 ± 5.29 | 93.41 ± 12.46 | 1.04 |
| Lysine | 381.07 ± 42.45 | 556.65 ± 38.21 | 1.46* |
| Putrescine | 2515.82 ± 205.19 | 2522.32 ± 353.67 | 1 |
| Cadaverine | 155.54 ± 17.45 | 142.66 ± 25.71 | 0.92 |
| Tyrosine | 660.66 ± 42.99 | 823.07 ± 25.29 | 1.25* |
| Methionine | 107.89 ± 5.54 | 188.78 ± 7.72 | 1.75*** |
| Valine | 878.74 ± 66.44 | 2086.18 ± 264.98 | 2.37** |
| Serotonin | 160.85 ± 10.77 | 245.94 ± 28.26 | 1.53* |
| Tyramine | 778.52 ± 206.94 | 439.33 ± 52.4 | 0.56 |
| Isoleucine | 119.28 ± 8.41 | 174.39 ± 23.57 | 1.46 |
| Leucine | 367.16 ± 35.78 | 661.66 ± 36.31 | 1.8*** |
| Phenylalanine | 407.3 ± 40.16 | 681.94 ± 48.52 | 1.67** |
| Tryptophan | 370.82 ± 32.98 | 436.24 ± 48.06 | 1.18 |
| Nicotianamine | 304.45 ± 37.7 | 287.94 ± 51.46 | 0.95 |
| 2’-Deoxymugineic acid | 6000.55 ± 312.53 | 9360.81 ± 1203.27 | 1.56* |

| Metabolite | 22:00 Collection time | | |
| --- | --- | --- | --- |
|  | +Fe | -Fe | IF |
| 4-hydroxy-proline | 19.21 ± 1.19 | 25.58 ± 1.79 | 1.33* |
| Histidine | 796.93 ± 67.06 | 1016.91 ± 98.99 | 1.28 |
| Asparagine | 8193.28 ± 1813.87 | 6575.9 ± 1085.36 | 0.8 |
| Arginine | 699.94 ± 58.58 | 878.86 ± 201.45 | 1.26 |
| Taurine | 15.05 ± 0.82 | 21.99 ± 1.74 | 1.46** |
| Serine | 3246.21 ± 343.6 | 3799.33 ± 403.52 | 1.17 |
| Glutamine | 24507.38 ± 7432.59 | 15796.32 ± 2917.83 | 0.64 |
| Homoserine | 347.51 ± 19.71 | 375.71 ± 23.19 | 1.08 |
| Glycine | 498.64 ± 45.11 | 519.35 ± 14.85 | 1.04 |
| Aspartate | 23397.5 ± 1593.1 | 23134.02 ± 3037.63 | 0.99 |
| Citrulline | 52.1 ± 4.17 | 61.56 ± 4.86 | 1.18 |
| Glutamate | 22497.29 ± 2433.92 | 21133.9 ± 1933.72 | 0.94 |
| beta-Alanine | 592.65 ± 43.32 | 1066.8 ± 219.73 | 1.8 |
| Threonine | 1427.42 ± 158.77 | 1685.03 ± 199.29 | 1.18 |
| Alanine | 3900.28 ± 858.85 | 2810.74 ± 336.99 | 0.72 |
| GABA | 2111.6 ± 472.71 | 2407.6 ± 342.98 | 1.14 |
| Proline | 787.55 ± 233.05 | 631.69 ± 85.89 | 0.8 |
| Cysteine | 351.97 ± 15.66 | 475.96 ± 23.55 | 1.35** |
| Ornithine | 36.85 ± 2.54 | 53.94 ± 15.14 | 1.46 |
| Octopamine | 88.75 ± 4.62 | 105.59 ± 8.25 | 1.19 |
| Lysine | 556.73 ± 49.61 | 780.54 ± 127.2 | 1.4 |
| Putrescine | 2564.01 ± 211.29 | 2363.73 ± 241.11 | 0.92 |
| Cadaverine | 158.1 ± 10.75 | 172.4 ± 9.9 | 1.09 |
| Tyrosine | 762.58 ± 17.68 | 869.05 ± 38.73 | 1.14* |
| Methionine | 134.63 ± 12.23 | 146.05 ± 14.37 | 1.08 |
| Valine | 1464.67 ± 213.98 | 2074.5 ± 419.83 | 1.42 |
| Serotonin | 135.01 ± 21.08 | 287.5 ± 33.9 | 2.13** |
| Tyramine | 416.6 ± 65.76 | 606.91 ± 87.83 | 1.46 |
| Isoleucine | 102.75 ± 19.2 | 210.96 ± 29.64 | 2.05* |
| Leucine | 641.95 ± 72.06 | 830.91 ± 147.46 | 1.29 |
| Phenylalanine | 617.65 ± 46.78 | 790.59 ± 148.63 | 1.28 |
| Tryptophan | 487.61 ± 17.25 | 692.12 ± 89.31 | 1.42 |
| Nicotianamine | 480.98 ± 101.39 | 519.87 ± 62.14 | 1.08 |
| 2’-Deoxymugineic acid | 6471.03 ± 893.08 | 10476.31 ± 1553.83 | 1.62 |

**Table S2B** **Iron deficiency associated induction of root amines concentrations.** Induction factor (IF) defined as the +Fe/ -Fe ratio of mean concentrations in the roots of 5 biological replicates. ↑↑: strongly upregulated (*p* < 0.05, IF ≥ 2), ↑: upregulated (*p* < 0.05, 1.5 < IF < 2), (↑): weakly upregulated (*p* < 0.05, 1 < IF < 1.5), -: no change (*p* ≥ 0.05). *p-*values calculated from Student’s t-tests performed between treatments within collection times.

| Metabolite | Collection time | | | |
| --- | --- | --- | --- | --- |
|  | 6:00 | 11:00 | 18:00 | 22:00 |
| **α-Ketoglutarate family** |  |  |  |  |
| 4-hydroxy-proline | - | - | - | (↑) |
| GABA | - | - | - | - |
| Glutamate | - | - | - | - |
| Glutamine | - | (↑) | ↑↑ | - |
| Histidine | - | (↑) | ↑ | - |
| Proline | ↑ | ↑ | ↑↑ | - |
| **Oxaloacetate/aspartate family** |  |  |  |  |
| Asparagine | - | - | ↑ | - |
| Aspartate | - | - | - | - |
| Homoserine | - | - | - | - |
| Isoleucine | ↑↑ | ↑ | - | ↑↑ |
| Lysine | - | ↑ | (↑) | - |
| Methionine | ↑ | ↑ | ↑ | - |
| Threonine | (↑) | (↑) | ↑ | - |
| **3-Phosphoglycerates** |  |  |  |  |
| Cysteine | (↑) | (↑) | - | (↑) |
| Glycine | (↑) | (↑) | ↑ | - |
| Serine | (↑) | (↑) | ↑ | - |
| Taurine | - | (↑) | - | (↑) |
| **Pyruvates** |  |  |  |  |
| β-Alanine | ↑ | ↑↑ | ↑↑ | - |
| Alanine | - | - | ↑↑ | - |
| Leucine | (↑) | ↑↑ | ↑ | - |
| Valine | ↑ | ↑↑ | ↑↑ | - |
| **DMA biosynthesis** |  |  |  |  |
| Nicotianamine | - | - | - | - |
| 2'-Deoxymugineic acid | - | ↑ | ↑ | - |
| **Aromatic amino acids** |  |  |  |  |
| Octopamine | - | - | - | - |
| Phenylalanine | - | ↑ | ↑ | - |
| Serotonin | ↑ | ↑ | ↑ | ↑↑ |
| Tryptophan | (↑) | ↑ | - | - |
| Tyramine | - | - | - | - |
| Tyrosine | (↑) | (↑) | (↑) | (↑) |
| **Urea Cycle/ Citrulline Nitric Oxide Cycle** |  |  |  |  |
| Arginine | ↑ | ↑↑ | - | - |
| Citrulline | (↑) | - | (↑) | - |
| Ornithine | - | - | - | - |
