# Supplementary material for: Diurnal Changes in Transcript and Metabolite Levels during the Iron Deficiency Response of Rice
Source: Rice (N Y). 2017 Apr 20;10:14. doi: 10.1186/s12284-017-0152-7 (PMC5398970; doi:10.1186/s12284-017-0152-7)
Supplement: Supplementary file 3 — Levels of 3-P-glycerate in Fe-sufficient and Fe-deficient rice roots quantified by LC-qTOF-MS. Figure S2. Levels of ascorbic acid in Fe-sufficient and Fe-deficient rice roots quantified by LC-qTOF-MS. (DOCX 117 kb) [file 12284_2017_152_MOESM3_ESM.docx]

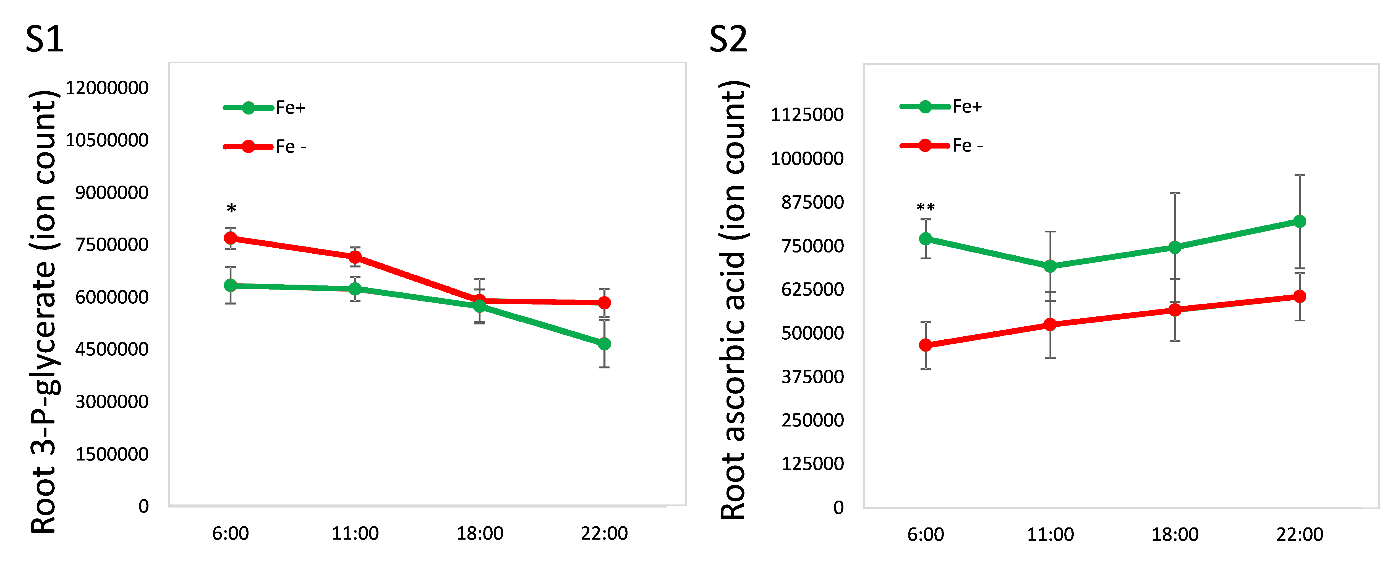


**Figures S1 and S2 Levels of 3-P-glycerate and ascorbic acid in Fe-sufficient and Fe-deficient rice roots.** Root levels of **S1**) 3-P-glycerate and **S2**) Ascorbic acid from Fe-sufficient (green) and Fe-deficient (red) rice plants were quantified by RP LC-qTOF-MS. Values are presented as means ± SE of 9 biological replicates. Significant differences are indicated by * for *p* < 0.05 and ** for *p* < 0.01 by Student’s t-tests performed between treatments within collection times.

A LC 1200 series infinity pump with autosampler and heated column compartment coupled to a 6520 series Accurate-Mass Quadrupole Time-Of-Flight (qTOF) MS (Agilent Technologies Inc.) was used for liquid chromatography quadrupole time-of-flight mass spectrometry (LC-qTOF-MS) analyses. During LC-MS analyses, the aqueous mobile phase, solvent ‘A’, was prepared as a 0.1% (v/v) formic acid (FA) in dH2O, and the organic mobile phase, solvent ‘B’, was prepared as 0.1% (v/v) FA in acetonitrile. Chromatography was performed using the gradient:

| Time (min) | A [%] | B [%] | Flow rate (µL/min) |
| --- | --- | --- | --- |
| 0.00 | 99 | 1 | 300 |
| 2.00 | 99 | 1 | 300 |
| 9.00 | 85 | 15 | 300 |
| 14.00 | 70 | 30 | 300 |
| 14.10 | 99 | 1 | 300 |
| 19.00 | 99 | 1 | 300 |

For LC-qTOF-MS analyses, MassHunter Profinder vB.06 (Agilent Technologies Inc.) was used to extract compound *m/z* and retention time (RT) which were then analysed using the Mass Profiler Professional software package v12.0 (Agilent Technologies Inc.). Putative identification of metabolites was performed based on the three assessment criteria: 1) accurate mass match of mono-isotopic peak (difference < 5 ppm), 2) RT match (± 20 s), and 3) match of isotopic peak profiles.
