# Supplementary material for: Diurnal Changes in Transcript and Metabolite Levels during the Iron Deficiency Response of Rice
Source: Rice (N Y). 2017 Apr 20;10:14. doi: 10.1186/s12284-017-0152-7 (PMC5398970; doi:10.1186/s12284-017-0152-7)
Supplement: Supplementary file 4 — Induction of precursors to methionine and DMA biosynthesis within the rice Fe deficiency response. (DOCX 831 kb) [file 12284_2017_152_MOESM4_ESM.docx]

**Figure S3 Induction of precursors to methionine and DMA biosynthesis within the rice Fe deficiency response.** Heatmap (ranging from 0–2.0 as white–red) of log_2_ transformed Fe deficiency associated induction of metabolite concentrations upstream of methionine and DMA biosynthesis.
