# Supplementary material for: Diurnal Changes in Transcript and Metabolite Levels during the Iron Deficiency Response of Rice
Source: Rice (N Y). 2017 Apr 20;10:14. doi: 10.1186/s12284-017-0152-7 (PMC5398970; doi:10.1186/s12284-017-0152-7)
Supplement: Supplementary file 5 — Iron replete hydroponic growth solution. Table S4. Primers used for qRT-PCR analyses. (DOCX 14 kb) [file 12284_2017_152_MOESM5_ESM.docx]

# Table S3 Iron replete hydroponic growth solution (pH 5.5).

| Salts | Final concentration (mM) |
| --- | --- |
| NH_4_NO_3_ | 5.0 |
| KNO_3_ | 5.0 |
| Ca(NO_3_)_2_•4H_2_O | 2.0 |
| MgSO_4_•7H_2_O | 2.0 |
| KH_2_PO_4_ | 0.1 |
|  | Final concentration (µM) |
| NaFe^3+^EDTA | 50.0 |
| H_3_BO_3_ | 50.0 |
| MnCl_2_•4H_2_O | 5.0 |
| ZnSO_4_•7H_2_O | 5.0 |
| CuSO_4_•5H_2_O | 0.5 |
| Na_2_MoO_3_ | 0.1 |

# Table S4 Primers used for qRT-PCR analyses.

| Gene | Primer | Primer sequence (5’–3’) |
| --- | --- | --- |
| *OsActin* | F Primer | GAAGATCACTGCCTTGCTCC |
|  | R Primer | CGATAACAGCTCCTCTTGGC |
| *OsGAPDH* | F Primer | GGGCTGCTAGCTTCAACATC |
|  | R Primer | TTGATTGCAGCCTTGATCTG |
| *OsELF1* | F Primer | ATCTGGGAAATCATCGGTTCTG |
|  | R Primer | AGATCGTCCACAATGGTCATCA |
| *OsNAS1* | F Primer | GTCATCGTCGCCCGCAAG |
|  | R Primer | ACTCTTCTGCCTTCTCAACCG |
| *OsNAS2* | F Primer | GTCATCGTCGCTCGCAAG |
|  | R Primer | GAACTCTTCCGCCTTCTGG |
| *OsNAS3* | F Primer | ATCAACTCCGTCATCATC |
|  | R Primer | CATGTCCTCCATCTTCTG |
| *OsNAAT1* | F Primer | GTCCTCACAAGCCCGAAG |
|  | R Primer | TTCATTCCCAGCACACTCC |
| *OsDMAS1* | F Primer | CCAATCCAAGGGCAAGAC |
|  | R Primer | ACGATGTCCAGGTTCTCC |
| *OsTOM1* | F Primer | GGCGTAGGGCTTGTTGTG |
|  | R Primer | TCTTTATGCTTATGAAGTGTCTCC |
| *OsYSL15* | F Primer | GGAACAAGATGAACAAGAAGGAG |
|  | R Primer | CGAGAGCAAGGATAGAAGAAGG |
